# Variation in zygotic CRISPR/Cas9 gene editing outcomes generates novel reporter and deletion alleles at the *Gdf11* locus

**DOI:** 10.1038/s41598-019-54766-y

**Published:** 2019-12-09

**Authors:** Jill M. Goldstein, Austin Valido, Jordan P. Lewandowski, Ryan G. Walker, Melanie J. Mills, Kathleen A. Messemer, Paul Besseling, Kyu Ha Lee, Samuel J. Wattrus, Miook Cho, Richard T. Lee, Amy J. Wagers

**Affiliations:** 1 Department of Stem Cell and Regenerative Biology, Harvard University, Cambridge, MA 02138 USA; 2 Harvard Stem Cell Institute, Cambridge, MA 02138 USA; 3 Paul F. Glenn Center for the Biology of Aging, Harvard Medical School, Boston, MA 02215 USA; 4 Department of Nutrition, Harvard T.H. Chan School of Public Health, Boston, MA 02115 USA; 5 Section on Islet Cell and Regenerative Biology, Joslin Diabetes Center, Boston, MA 02215 USA

**Keywords:** Biological techniques, Genetics

## Abstract

Recent advances in CRISPR/Cas gene editing technology have significantly expanded the possibilities and accelerated the pace of creating genetically engineered animal models. However, CRISPR/Cas-based strategies designed to precisely edit the genome can often yield unintended outcomes. Here, we report the use of zygotic CRISPR/Cas9 injections to generate a knock-in GFP reporter mouse at the *Gdf11* locus. Phenotypic and genomic characterization of founder animals from these injections revealed a subset that contained the correct targeting event and exhibited GFP expression that, within the hematopoietic system, was restricted predominantly to lymphoid cells. Yet, in another subset of founder mice, we detected aberrant integration events at the target site that dramatically and inaccurately shifted hematopoietic GFP expression from the lymphoid to the myeloid lineage. Additionally, we recovered multiple *Gdf11* deletion alleles that modified the C-terminus of the GDF11 protein. When bred to homozygosity, most of these alleles recapitulated skeletal phenotypes reported previously for *Gdf11* knockout mice, suggesting that these represent null alleles. However, we also recovered one *Gdf11* deletion allele that encodes a novel GDF11 variant protein (“GDF11-WE”) predicted to contain two additional amino acids (tryptophan (W) and glutamic acid (E)) at the C-terminus of the mature ligand. Unlike the other *Gdf11* deletion alleles recovered in this study, homozygosity for the *Gdf11*^*WE*^ allele did not phenocopy *Gdf11* knockout skeletal phenotypes. Further investigation using *in vivo* and *in vitro* approaches demonstrated that GDF11-WE retains substantial physiological function, indicating that GDF11 can tolerate at least some modifications of its C-terminus and providing unexpected insights into its biochemical activities. Altogether, our study confirms that one-step zygotic injections of CRISPR/Cas gene editing complexes provide a quick and powerful tool to generate gene-modified mouse models. Moreover, our findings underscore the critical importance of thorough characterization and validation of any modified alleles generated by CRISPR, as unintended on-target effects that fail to be detected by simple PCR screening can produce substantially altered phenotypic readouts.

## Introduction

Efforts to engineer genetic mouse models have progressed rapidly in recent years due in part to advances in CRISPR/Cas (Clustered Regularly Interspaced Palindromic Repeats/CRISPR associated protein) technology^1–3^. Type II CRISPR/Cas systems have been adapted to function as gene editing tools, whereby programmable single guide RNAs (sgRNAs) escort the endonuclease Cas9 to a target DNA sequence and enable the introduction of site-specific DNA double-strand breaks (DSBs)^4–10^. Absent a homologous DNA template, cells repair these DSBs through non-homologous end joining (NHEJ), an error-prone mechanism that leads to the formation of insertions and deletions (indels) that can disrupt the normal reading frame of protein-coding genes. In contrast, when homologous DNA templates are present, cells can repair DSBs via homology-directed repair (HDR), which allows for the targeted integration of specific genomic sequences at the cut site^1–3^. Unlike traditional strategies that require time-consuming gene-targeting and selection in embryonic stem cells prior to blastocyst injection^11^, direct injection of CRISPR/Cas9 gene editing components into mouse zygotes can generate gene-modified mice within as little as three weeks and in any of a number of genetic backgrounds^12–14^.

Yet, despite the advantages of CRISPR/Cas9 systems for generating precise gene editing outcomes, reports of off-target effects, genomic rearrangements, and large deletions have introduced concerns regarding the use of CRISPR/Cas9 as a high-fidelity genome modification technology^15–17^. Indeed, several investigators have noted allelic complexity and/or mosaicism in founder animals generated by zygotic injection of CRISPR/Cas9 complexes^18–21^. Yet, while CRISPR/Cas9 zygotic injection has been applied in many studies to generate gene-modified mouse models^12,13,20,22–25^, relatively few of these studies have specifically reported on the potential vulnerabilities of this approach for establishing *in vivo* research models, and community norms for validating CRISPR/Cas engineered animals have not been clearly defined. Here, we report the generation of a transgenic reporter mouse at the *Gdf11* locus using CRISPR/Cas technology, highlighting both the effectiveness and the complexity of gene editing outcomes resulting from this approach and identifying effective strategies to decode the varied allelic outcomes.

We sought to target the mouse *Gdf11* locus, which encodes a secreted TGF-β ligand that is essential for postnatal life. *Gdf11* knockout mice do not survive beyond 24 hours after birth^26,27^ and display multiple developmental phenotypes^28–32^, including homeotic skeletal transformations, ectopic ribs, tail malformations^26,33^, and craniofacial/palatal defects^34–36^. *Gdf11* heterozygous mice are viable and exhibit haploinsufficient developmental phenotypes, including the presence of an additional rib^26^. While less is understood about the role of GDF11 in adulthood, several groups have investigated its effects on aging in mice and humans. However, technical challenges in specifically discriminating GDF11 from other closely related TGF-β molecules (e.g. GDF8, also known as Myostatin) have contributed to confusion regarding the direction of change with age of GDF11 levels^37–42^. Motivated by this lack of clarity, along with the insufficiency of molecular tools to specifically assay GDF11 production *in vivo*, we decided to create a knock-in GFP reporter mouse at the *Gdf11* locus using zygotic CRISPR/Cas9 injections. This reporter mouse would enable direct analysis of *Gdf11* expression at the single cell level, revealing how both *Gdf11* expression and the frequencies of *Gdf11*-expressing cells may change during aging.

Our initial efforts to generate *Gdf11*-IRES-GFP reporter mice using zygotic CRISPR/Cas9 injections produced 5 independent *Gdf11*-IRES-GFP mouse lines from 4 founder animals, and all of these founders screened positive by PCR for the *Gdf11*-IRES-GFP reporter at the target locus. While 3 of these lines contained the correctly targeted *Gdf11*-IRES-GFP transgene, upon deeper analysis, 2 lines were subsequently found to contain aberrant integrations at the target site. The 2 incorrectly targeted lines exhibited a strikingly different pattern of GFP expression within hematopoietic lineage cells (a primary source of *Gdf11* expression^37^) relative to the 3 correctly targeted lines. Profiling of *Gdf11*-IRES-GFP expression within hematopoietic cells from the correctly targeted GFP mouse lines revealed relatively static *Gdf11*-IRES-GFP expression within T cells and more dynamic changes within B cells during aging. We also recovered 4 novel alleles containing multinucleotide deletions at the target *Gdf11* locus. These deletions are predicted to disrupt the endogenous stop codon and induce partial translation of the 3′UTR. When bred to homozygosity, 3 of these alleles recapitulated the skeletal defects reported for *Gdf11* knockout mice^26,33^. Interestingly, one of these alleles did not induce these same skeletal defects, and mice heterozygous or homozygous for this variant allele remained viable through adulthood. These findings suggest that this GDF11 variant (termed GDF11-WE due the addition of a tryptophan (W) and a glutamic acid (E) at the C-terminus) retains substantial function *in vivo* and provides unexpected insights into the biology of GDF11. Altogether, this work emphasizes that while CRISPR/Cas9-based approaches to generate gene-modified mouse models offer many advantages, care must be taken to validate that on-target editing events occur as intended, especially since aberrant integration events at the target site may not be detected by PCR-based approaches. Furthermore, this work identifies effective strategies to discriminate such genomic “side effects”, some of which can provide useful biological insights, from intended sequence modifications.

## Results

### Generation of a *Gdf11* reporter construct and founder mice

We sought to target a fluorescent reporter gene to the mouse *Gdf11* locus using CRISPR/Cas9 and first tested this approach in cell culture. We began by designing single guide RNAs (sgRNAs) compatible with *Streptococcus pyogenes* Cas9 (spCas9) to target the mouse *Gdf11* locus. Based on the location of the protospacer adjacent motif (PAM) for each sgRNA, spCas9 was predicted to cut after the stop codon and at the beginning of the 3′UTR (Fig. S1A). To test the ability for each sgRNA to direct spCas9 to the intended genomic location, we transfected mouse C2C12 cells with an spCas9-2A-mCherry expression vector, together with individual sgRNA expression plasmids. Three days after transfection, we FACS-purified mCherry^+^ and mCherry^−^ cells (Fig. S1B) and PCR-amplified a 797-base pair (bp) amplicon surrounding the target sequence (Fig. S1C). T7 Endonuclease I (T7EI) mismatch repair assays^43^ indicated the introduction of indels at the target site for all four sgRNAs in the mCherry^+^ cells, confirming that each could effectively target the *Gdf11* locus (Fig. S1C). We also detected faint bands indicating indels at the target site in the mCherry^−^ cells (Fig. S1C), which likely reflects impurities in sorting the mCherry^−^ fraction or that this fraction included cells that had already expressed Cas9 and targeted the *Gdf11* locus, but had not yet robustly expressed mCherry.

**Figure 1:**
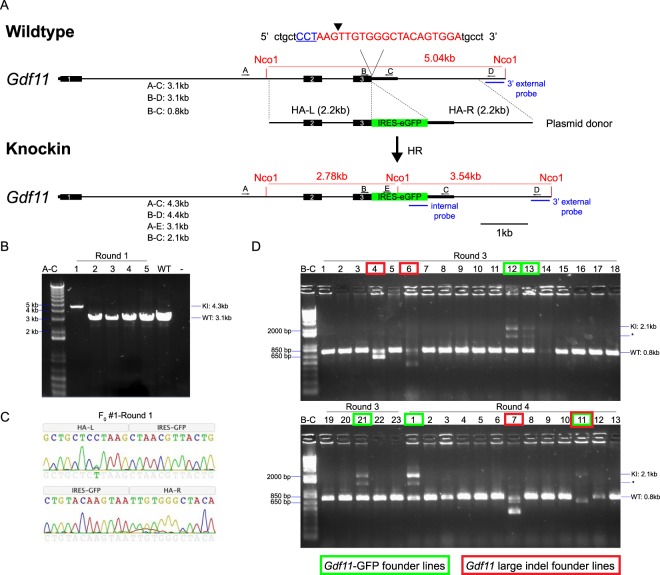
Generation of *Gdf11*-IRES-GFP knock-in reporter mice using CRISPR/Cas9. (**A)** Schematic of *Gdf11*-IRES-GFP targeting to the *Gdf11* locus. Blue underlined text indicates the protospacer adjacent motif (PAM) for sgRNA3. Red text indicates the target sequence for sgRNA3. Black arrowhead indicates the predicted cut site for sgRNA3. Primers used for PCR-based screening are designated as A, B, C, D, and E above each allele, and predicted amplicon sizes are listed beneath each allele. The location of NcoI restriction sites and Southern blot probe sequences are indicated in red and blue text, respectively. HA-L: Left homology arm. HA-R: Right homology arm. (**B**) PCR screening of 5 founder mice from Round #1 of injections using primer pair A–C. Expected size: WT = 3.1 kb; KI = 4.3 kb. Gel image is uncropped with the entirety of the captured image shown. (**C)** Chromatogram illustrating sequence of boundaries between *top:* left homology arm (HA-L) and IRES-GFP, and *bottom:* IRES-GFP and right homology arm (HA-R). (**D)** PCR screening of 36 founder mice from Rounds #3 and #4 of using primer pair B-C. Expected size: WT = 0.8 kb; KI = 2.1 kb; *Non-specific band. Green boxes indicate founder animals harboring the *Gdf11*-IRES-GFP knock-in allele. Red boxes indicate founder animals harboring large deletions in *Gdf11*. Gel image is uncropped. Positive and negative control reactions for PCR amplification were run on a separate gel, which is presented in Supplementary Fig. 12A.

We next developed a strategy to introduce a GFP reporter into the mouse *Gdf11* locus using HDR. As GDF11 is a secreted ligand whose activity requires multiple proteolytic processing events^44^, we decided against a direct protein fusion approach, which could cause fusion-related disruptions in GDF11 expression, stability or signaling capacity. Instead, we pursued a bicistronic approach to drive GFP expression from an internal ribosome entry site (IRES), a strategy used previously for the generation of analogous reporter lines^45–47^. We cloned a plasmid donor containing IRES-GFP sequences flanked by 2.2 kb homology arms (Fig. 1A) and transfected this construct into C2C12 cells together with spCas9-2A-mCherry and individual sgRNA expression plasmids. PCR analysis confirmed integration of the IRES-GFP template at the *Gdf11* locus (Fig. S1D). We further validated this genomic targeting event using Sanger sequencing, which also confirmed the integration of a silent mutation (C→T) that we included in the plasmid donor to mutate the PAM sequence and prevent re-cutting after integration of the donor template (Fig. S1E). Fluorescence microscopy analysis detected transfected C2C12 cells expressing both mCherry and GFP (Fig. S1F). Together, these results confirmed our approach for CRISPR/Cas9 targeting of an IRES-GFP reporter to the mouse *Gdf11* locus.

We then generated knock-in *Gdf11*-IRES-GFP mice using one-step zygotic injections. We performed 4 rounds of injections in C57BL/6J zygotes by delivering an *in vitro*-transcribed sgRNA, circular *Gdf11*-IRES-GFP plasmid donor template, and either *spCas9* mRNA (Rounds #1, 2, and 4) or spCas9 protein (Round #3). In total, we produced 57 live pups and 6 dead pups (Round #1: 5 live pups; Round #2: 16 live pups; Round #3: 23 live pups; Round #4: 13 live pups, 6 dead pups). All animals were screened for the targeted allele by PCR (primers A-C and B-C; Fig. 1A). Of the 6 dead pups, 4 lacked a tail (data not shown), which is consistent with the tailless phenotype reported previously for *Gdf11* knockout mice^26,33^. Sequencing of the *Gdf11* target site in DNA from the 4 tailless pups identified multiple indel mutations that disrupt the *Gdf11* stop codon and lead to extensive translation of the 3′UTR (pups A-D; Fig. S2C). Given that these pups recapitulate the tailless phenotype observed in *Gdf11* knockout mice^26,33^, we hypothesized that these mutations likely render GDF11 non-functional. We also detected multiple *Gdf11* indels in the 2 dead pups that did not lack tails (pups E-F; Fig. S2D). These mutations either eliminate 1–2 amino acids at the C-terminus or produce non-coding disruptions in the 3′UTR (Fig. S2D). Given that these pups do not phenocopy the tailless phenotype of the *Gdf11* null mouse, these indels may preserve the function of GDF11, although expression of some loss-of-function phenotypes in these zygotically-injected mice could also have been suppressed by genetic mosaicism.

### Characterization of progeny from zygotic CRISPR injections

Screening of the 57 live pups produced from our CRISPR/Cas injections using internal and knock-in specific PCR primers identified 6 founder animals harboring the *Gdf11*-IRES-GFP allele (Round #1: 1 founder; Round #2: 0 founders; Round #3: 3 founders; Round #4: 2 founders) (Figs. 1B,D and S2A,B). In founder #1 from Round #1, we only detected the presence of a knock-in-sized amplicon, suggesting biallelic targeting (Fig. 1B). Sanger sequencing of the knock-in sized amplicon from this founder (Fig. 1C) confirmed the presence of the silent mutation (C→T) included in the HDR template, but additionally showed a peak for the native C nucleotide on the chromatogram (Fig. 1C). These results indicate mosaicism of founder #1 from Round #1, as has been reported previously^18,19^. During our PCR screening, we also identified 4 mice with a discernably smaller amplicon than the wild type-sized amplicon (red boxes; Fig. 1D).

**Figure 2:**
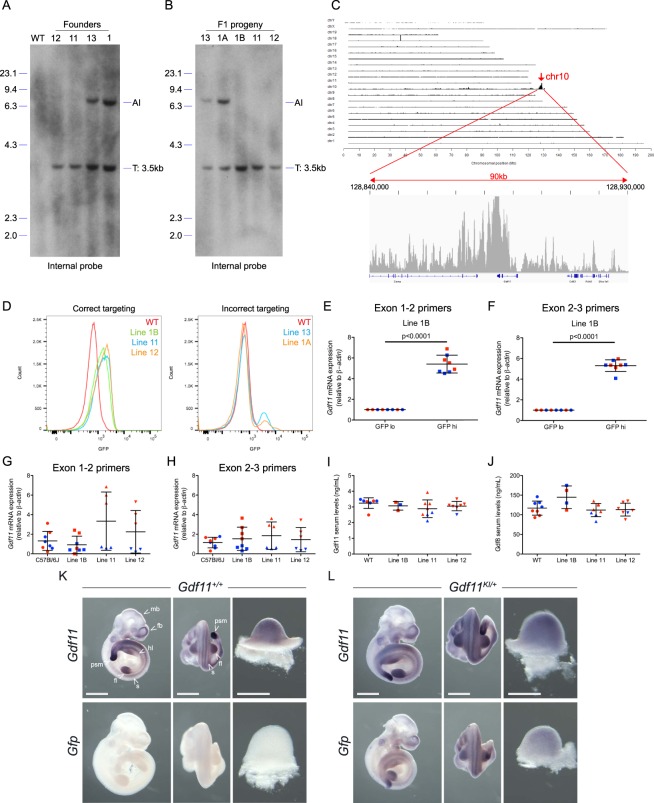
Validation of *Gdf11*-IRES-GFP knock-in reporter mouse lines. (**A**,**B**) Southern blot analysis of (**A**), *Gdf11-*IRES-GFP targeted founder mice and (**B**), *Gdf11-*IRES-GFP F1 progeny. Nco1-digested genomic DNA was hybridized with the internal probe. Expected fragment size: WT = n/a; T (targeted) = 3.5 kb. AI: Additional integration. Blot images were cropped to focus on the target bands. Uncropped blots are presented in Supplementary Fig. 12B,C. (**C**) TLA sequencing coverage and analysis plots from line 1B using outward facing primers residing in the GFP transgene. (**D**) Flow cytometry analysis of GFP expression in live (7AAD^−^) peripheral blood cells in *left*: mice exhibiting correct targeting (lines 1B, 11 and 12) and *right:* mice exhibiting incorrect targeting (lines 1A and 13). (**E,F**) Real time PCR analysis of *Gdf11* levels in FACS-purified GFP^high^ and GFP^low^ splenocytes from line 1B using (**E**), primers spanning exons 1-2 and (**F**), primers spanning exons 2–3. *β*-*actin* was used as a housekeeping gene. Transcript levels were normalized to levels in GFP^low^ splenocytes. N = 4 males (blue), 4 females (red). Data are presented as individual data points overlaid with mean ± SD. (**G,H**) Real time PCR analysis of *Gdf11* levels in whole spleen from correctly targeted lines (1B, 11 and 12) and age- and sex-matched C57BL/6J mice. Relative *Gdf11* expression levels were assayed using (**G**), primers spanning exons 1–2 and (**H)**, primers spanning exons 2–3. *β*-*actin* was used as a housekeeping gene. Transcript levels were normalized to levels in C57BL/6J mice. N = 3–4 males (blue), 3–4 females (red). Data are presented as individual data points overlaid with mean ± SD. (**I,J**), Quantification of (**I**), GDF11 protein levels, and (**J)**, GDF8 protein levels, in serum from correctly targeted lines (1B, 11 and 12) and age- and sex-matched C57BL/6J mice. (**K,L**) Whole mount *in situ* hybridization for *Gdf11* (top) and *Gfp* (bottom) in E10.5 *Gdf11*^+/+^ and *Gdf11*^KI/+^ embryos from line 1B. For each embryo, the right-most images show the dissected forelimb. mb: midbrain, fb: forebrain, psm: pre-somitic mesoderm, fl: forelimb, hl: hindlimb, s: somite. Scale bar: 0.5 mm.

Collectively, 4 of the 6 *Gdf11*-IRES-GFP targeted founders survived to adulthood, and we used these mice to establish independent lines for further validation studies. Southern blot analysis revealed that while all 4 founders contained the *Gdf11*-IRES-GFP at the intended locus, founders #1 and #13 also contained an additional integration (AI) event (Figs. 2A, S3A and S12B). When founder #1 was outbred to a C57BL/6J mouse, the F1 progeny either contained only the correctly targeted allele (see mouse 1B in Fig. 2B) or both the targeted allele and an additional integration event (see mouse 1A in Fig. 2B). When founder #13 was outbred to a C57BL/6J mouse, the additional integration event segregated with the correctly targeted event (Figs. 2B, S3B and S12C). Next, we performed Targeted Locus Amplification (TLA) sequencing^48,49^ using two independent primer sets to identify the genomic location of each targeting event. TLA results confirmed that line 1B contained the correctly integrated IRES-GFP transgene at the *Gdf11* locus (Figs. 2C and S3C). TLA sequencing also demonstrated targeting at the *Gdf11* locus in lines #1A and #13; however, the integration events in these lines incorporated the intended donor sequence along with the plasmid backbone, likely as a concatemer (data not shown). Thus, the additional integrations in lines #1A and #13 were not off-target integrations of the transgene, but rather aberrant integration events at the intended *Gdf11* target site. In summary, while all founders that screened positive by PCR were indeed targeted at the *Gdf11* locus, only a fraction of these alleles contained the correct insertion of the IRES-GFP cassette behind the *Gdf11* coding sequence.

### The knock-in *Gdf11*-GFP gene accurately reports *Gdf11* expression in correctly targeted lines

**Figure 3:**
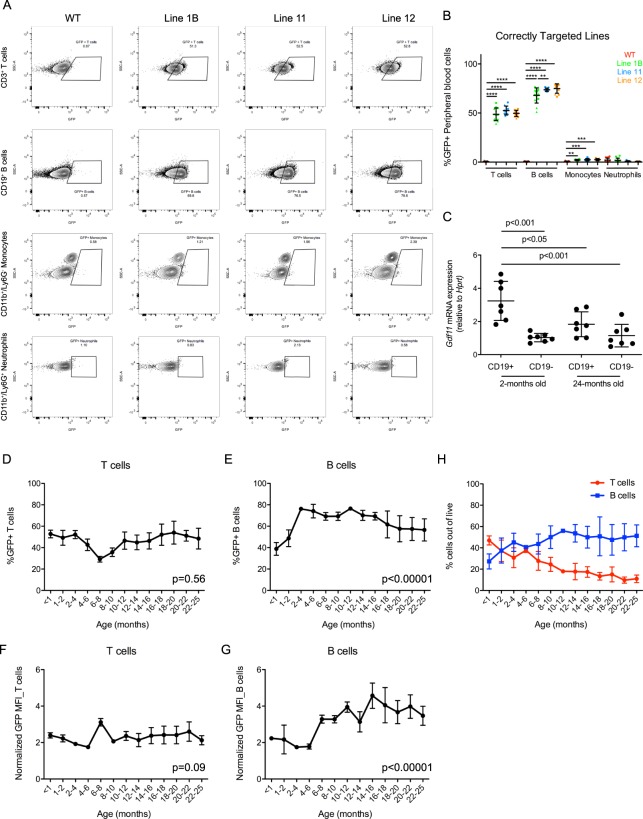
*Gdf11*-IRES-GFP expression is primarily detected within T and B lymphocytes of the peripheral blood. (**A**) Representative flow cytometry analysis of GFP expression within CD3^+^ T cells, CD19^+^ B cells, CD11b^+^/Ly6G^−^ monocytes and CD11b^+^/Ly6G^+^ neutrophils from peripheral blood. (**B**) Quantification of GFP+ T cells, B cells, monocytes and neutrophils in 2 month old mice from lines 1B, 11 and 12 and WT controls. N = 3–8 males and 3–8 females per genotype. Circles: males. Triangles: Females. Individual data points overlaid with mean ± SD. (**C**) Real time PCR analysis of *Gdf11* levels in CD19+ and CD19- splenic cells from young (2-month old) and aged (24-month old) mice. *Hprt* was used as a housekeeping gene. (**D,E**) Quantification of (**D**), GFP + peripheral blood T cells and (**E**), GFP + peripheral blood B cells within heterozygous mice from line 1B during aging. (**F,G**) Quantification of GFP mean fluorescence intensity within **F**, peripheral blood T cells and (**G**), peripheral blood B cells in heterozygous mice from line 1B during aging. Mean fluorescence intensity (MFI) values normalized to wild type mice for each timepoint. (**H**) Quantification of total T cell frequency (red) and B cell frequency (blue) out of live peripheral blood cells during aging. N = 25 males and 19 females. Data points represent mean with error bars denoting SEM.

We next performed flow cytometry to detect GFP fluorescence within peripheral blood cells of progeny from the 5 different *Gdf11*-IRES-GFP alleles. Interestingly, the pattern of GFP fluorescence varied substantially between the 2 categories of targeted mice (Fig. 2D). In the correctly targeted lines (line #1B, #11 and #12), GFP fluorescence was detected primarily in T and B lymphocytes, with minimal fluorescence in monocytes and neutrophils (Fig. 3A,B). These results are consistent with publicly available datasets reporting enrichment of *Gdf11* expression in lymphocytes relative to other hematopoietic cell types^50–52^. Our own analysis further confirmed enrichment of *Gdf11* mRNA within splenic CD19+ B-lineage cells relative to CD19- cells in 2-month old mice (Fig. 3C). Conversely, the 2 incorrectly targeted lines (line #1A, #13) exhibited robust GFP fluorescence in monocytes and neutrophils and minimal GFP fluorescence in lymphoid cells (Fig. S4A,B). While we cannot explain the molecular mechanisms underlying this striking lymphoid to myeloid switch in GFP expression in the incorrectly targeted GFP reporter mouse lines, these findings emphasize the need to rigorously validate new CRISPR-generated mouse lines using multiple molecular techniques, as aberrant integration events at the target site can dramatically alter reporter gene expression and phenotypic readouts.

Focusing our further efforts on only the correctly targeted *Gdf11*-IRES-GFP mouse lines, we performed additional studies to determine whether GFP fluorescence accurately reports *Gdf11* mRNA expression within these animals. To this end, we FACS-purified GFP^high^ and GFP^low^ splenocytes from homozygous animals and performed real-time PCR analysis for *Gdf11* using two independent primer sets. We observed approximately 5-fold greater levels of *Gdf11* mRNA in GFP^high^ relative to GFP^low^ splenocytes for all 3 *Gdf11-*IRES-GFP reporter lines (Figs. 2E,F and S3D–G). Next, to determine if IRES-GFP integration altered *Gdf11* expression in the reporter mice, we compared splenic *Gdf11* mRNA and serum GDF11 protein levels in homozygous reporter animals to age- and sex-matched C57BL/6J controls. To specifically measure GDF11 protein levels, we employed a recently developed liquid chromatography tandem mass spectrometry assay that selectively discriminates between GDF11 and GDF8 by measuring two divergent peptide fragments^53^. Splenic *Gdf11* mRNA expression (Fig. 2G,H) and serum GDF11 protein levels (Fig. 2I) were not significantly different among the 3 reporter lines and C57BL/6J controls, indicating that introduction of the IRES-GFP reporter sequence did not perturb endogenous GDF11 production. GDF8 serum levels were also unchanged in the reporter mice (Fig. 2J). Lastly, we analyzed the spatial expression pattern of *Gdf11* and *Gfp* mRNA in reporter animals by *in situ* hybridization and compared these results to the pattern of endogenous *Gdf11* mRNA expression in age-matched C57BL/6J mice. Patterns of *Gdf11* expression were indistinguishable in C57BL/6J and *Gdf11*-IRES-GFP line #1B heterozygous embryos at E10.5 (Fig. 2K,L) and consistent with published data indicating enrichment of *Gdf11* expression in somites, tail tip, limb bud, and forebrain at this developmental stage^26,27^. Hybridization with antisense *Gfp* probes further revealed an expression pattern similar to that of *Gdf11* in *Gdf11*-IRES-GFP line #1B heterozygous embryos and a lack of signal in C57BL/6J embryos (Fig. 2K,L), confirming that *Gfp* expression recapitulates *Gdf11* expression.

### Analysis of *Gdf11*-GFP fluorescence reveals dynamic expression of *Gdf11* during aging

We utilized our *Gdf11*-IRES-GFP line #1B animals to evaluate the dynamics of *Gdf11* mRNA expression during aging. Given that hematopoietic lineage cells express high levels of *Gdf11* during youth relative to other tissues^37^, we chose to profile *Gdf11* expression in discrete subsets of peripheral blood cells from 3 weeks of age to 25 months of age. Approximately 40–50% of T cells in *Gdf11*-IRES-GFP mice exhibited detectable GFP signal throughout most of life with a transient drop to ~20% of T cells at 6–8 months of age (Fig. 3D). The mean fluorescence intensity of GFP, reflecting the relative level of *Gdf11* expression in individual cells, was also largely constant in T cells during aging, with a slight spike at the 6–8 month timepoint (Fig. 3F). These results indicate that both the level of *Gdf11* expression and the proportion of *Gdf11*-expressing peripheral T cells remain largely steady during aging. However, as T cell frequency among peripheral blood mononuclear cells (PBMCs) decreases from ~45% at 3 weeks of age to ~10% at 22–24 months of age, while B cell frequency increases from ~25% at 3 weeks to ~51% at 22–24 months of age (Fig. 3H), it is likely that T cells become proportionately lesser contributors to hematopoietic production of GDF11 with increasing age.

The profile of *Gdf11*-IRES-GFP expression during aging was slightly different for B lymphocytes. From 3 weeks to 4 months of age, the frequency of GFP+ B cells increased from ~35% to ~70%, but then decreased after 4 months of age, eventually dropping to ~60% at 24 months of age (Fig. 3E). However, GFP mean fluorescence intensity showed an overall increase in B cells during aging (Fig. 3G). Thus, while the fraction of peripheral blood B cells that express *Gdf11* gradually decreases in adult mice after 4 months of age, those *Gdf11*-expressing B cells that remain in older animals appear to increase *Gdf11*-GFP expression. This change in *Gdf11*-GFP expression may compensate to some degree for the observed age-related loss of *Gdf11*-producing B cells; however, bulk analysis of *Gdf11* mRNA levels in splenic CD19+ B cells or CD19− non-B cells from young or aged mice using real-time PCR suggests that the total abundance of *Gdf11* mRNA in B lymphocytes still declines with age (Fig. 3C). These apparently divergent trajectories of *Gdf11* expression in aging B cells could reflect differences in the anatomical site of analysis (peripheral blood versus spleen), aging-related changes in *Gdf11* mRNA translational efficiency, or other as yet undiscovered regulatory mechanisms. Regardless, these data underscore the importance of applying single cell resolution, as achieved by flow cytometric analysis of the fractional representation of *Gdf11*-GFP-expressing B cells, to dissect the complexities of gene expression dynamics in aging lymphocyte subsets.

### *Gdf11* deletion alleles recapitulate developmental phenotypes of *Gdf11*^−/−^ embryos

**Figure 4:**
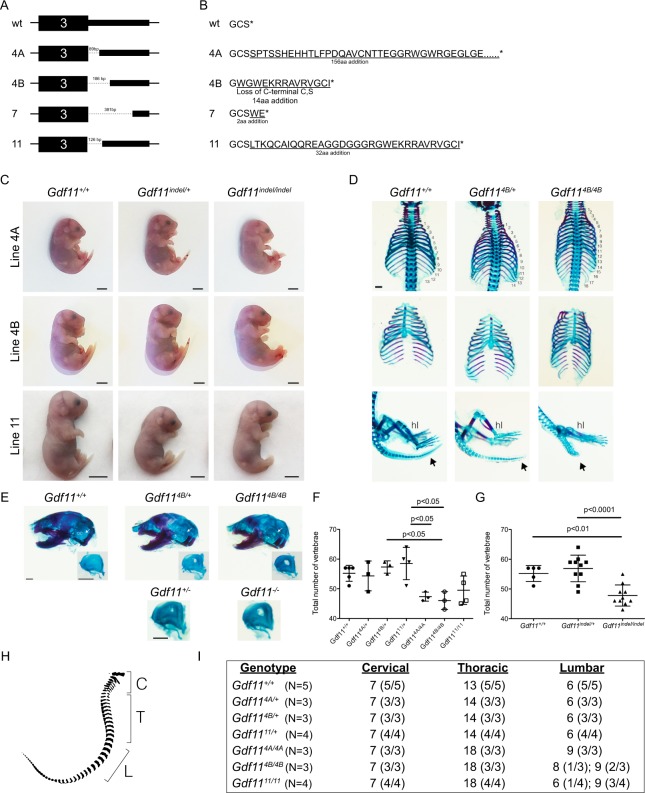
Novel *Gdf11* deletion alleles recapitulate skeletal defects observed in *Gdf11* knockout mice. (**A**) Schematic of *Gdf11* deletion alleles recovered from Rounds #3 and #4 of CRISPR injections. Dotted lines denote deleted region of the DNA sequence. Size of deletion noted above dotted line. (**B**) Schematic of exogenous amino acids added (underlined) to the C-terminal domain of GDF11 in the four *Gdf11* deletion alleles. Native amino acids are not underlined. *Stop codon. (**C**) Images of E17.5 or E18.5 embryos from lines 4A, 4B and 11 depicting loss of the tail in homozygous animals. (**D**) Skeletal preparation analysis of *Gdf11*^+/+^, *Gdf11*^4B/+^ and *Gdf11*^4B/4B^ embryos stained with Alcian Blue (to mark cartilage) and Alizarin Red (to mark bone). Numbers indicate thoracic vertebrae. Black arrow denotes tail, which is visibly shortened in the homozygote. hl: hindlimb. (**E**) *Top:* Skeletal preparations of skulls from *Gdf11*^+/+^, *Gdf11*^4B/+^ and *Gdf11*^4B/4B^ embryos stained with Alcian Blue and Alizarin Red. oc: otic capsule. White arrow denotes oc. Insets depict isolated oc. *Bottom:* Isolated oc from *Gdf11*^+/−^ and *Gdf11*^−/−^ and embryos. Scale bar: 1 mm. (**F,G**) Quantification of total number of vertebrae within embryos from **F**, each individual deletion allele, and (**G**), all deletion alleles pooled together. (**H**) Schematic of vertebrae depicting quantification of cervical (C), thoracic (T) and lumbar (L) vertebrae. (**I**) Quantification of the number of cervical, thoracic and lumbar vertebrae among the genotypes indicated.

Following analysis of the *Gdf11*-IRES-GFP knock-in reporter animals, we revisited the *Gdf11* deletion alleles that we also recovered after targeting of the *Gdf11* locus (red boxes; Fig. 1D). Founder #4 from Round #3 contained two distinct *Gdf11* deletion alleles that segregated in the F1 generation, termed 4A and 4B (data not shown). Sanger sequencing of all 4 deletion alleles recovered from 3 founders indicated deletions ranging in size from 89 bp up to 381 bp (Fig. 4A). *In silico* translation of these sequences yielded predicted peptide sequences with an additional 2–156 amino acids at the C-terminus of the protein due to partial translation of the 3′UTR (Fig. 4B). To evaluate the impacts of these novel deletion alleles, we assayed their effects on GDF11 function *in vivo*. Given that our zygotic injections produced several dead pups that lacked a tail and harbored frameshift mutations leading to extensive translation of the *Gdf11* 3′UTR, we hypothesized that these recovered deletion alleles would likewise induce skeletal abnormalities when bred to homozygosity. For each deletion line, we established timed matings between heterozygous mice and harvested E17.5 or E18.5 stage embryos. While wild type (*Gdf11*^+/+^) and heterozygous (*Gdf11*^*indel*/*+*^) embryos from lines 4A, 4B, and 11 exhibited an external tail, homozygous (*Gdf11*^*indel*/*indel*^) embryos from all 3 of these lines lacked a tail (Fig. 4C), similar to engineered *Gdf11* null mice^26,33^. Whole mount skeletal preparations from these embryos further confirmed the skeletal abnormalities associated with these alleles, as all heterozygous embryos contained one extra thoracic vertebra and all homozygous embryos contained five additional thoracic vertebrae relative to wild type controls (Fig. 4D,H,I). Altogether, embryos homozygous for deletion 4A, 4B or 11 showed a significant decrease in total vertebrae number (Fig. 4F,G), with expansions in the number of both thoracic (T) and lumbar (L) vertebrae (Fig. 4H,I). These skeletal phenotypes are consistent with previously reported phenotypes from *Gdf11* knockout mice^26,33^, which frequently exhibit a C7/T18/L8 pattern^26^, and strongly suggest that these 3 genomic deletions render the *Gdf11* allele non-functional.

Interestingly, during our analyses of the skeletons of these *Gdf11* indel mice, we also observed an additional skeletal phenotype within the otic capsule of the skull, a bone that surrounds the inner ear^54^. Both heterozygous and homozygous embryos exhibited a discernable hole within the otic capsule (Fig. 4E; top panels). This phenotype had not been reported previously for *Gdf11* null mice, thus prompting us to interrogate embryos heterozygous or homozygous for the previously generated *Gdf11* null allele^26,33^ for this phenotype. We identified this feature also in the previously described genetically engineered *Gdf11* heterozygous and knockout mice (Fig. 4E; bottom panels), confirming that this phenotype indeed arises due to the disruption of *Gdf11*.

In addition to skeletal phenotypes, *Gdf11* knockout mice exhibit a spectrum of renal defects, particularly unilateral and bilateral kidney agensis^29^. We therefore quantified kidney number in E18.5 stage *Gdf11*^+/+^, *Gdf11*^*indel*/*+*^, and *Gdf11*^*indel*/*indel*^ embryos from lines 4A and 11 to determine if kidney agenesis was also a feature of these newly generated *Gdf11* loss-of-function alleles. 100% of *Gdf11*^+/+^ embryos (5 out of 5 examined) exhibited two kidneys (Fig. S5A,B), as did most *Gdf11*^*indel*/*+*^ embryos. 4% (1 out of 25 examined) of *Gdf11*^*indel*/*+*^ embryos exhibited unilateral renal agenesis (Fig. S5A,B), consistent with previously reported results from *Gdf11* heterozygous mice^29^. In contrast, 63.6% (7 out of 11 analyzed) of *Gdf11*^*indel*/*indel*^ embryos exhibited bilateral renal agenesis and 36.4% (4 out of 11 analyzed) exhibited unilateral renal agenesis (Fig. S5A,B), again consistent with previous results in *Gdf11* knockout mice^29^. Taken together, these findings provide additional supportive evidence that the *Gdf11* deletion alleles recovered in this study represent null alleles of *Gdf11*.

### *Gdf11*^*WE*^ deletion allele generates a functional GDF11 protein variant

**Figure 5:**
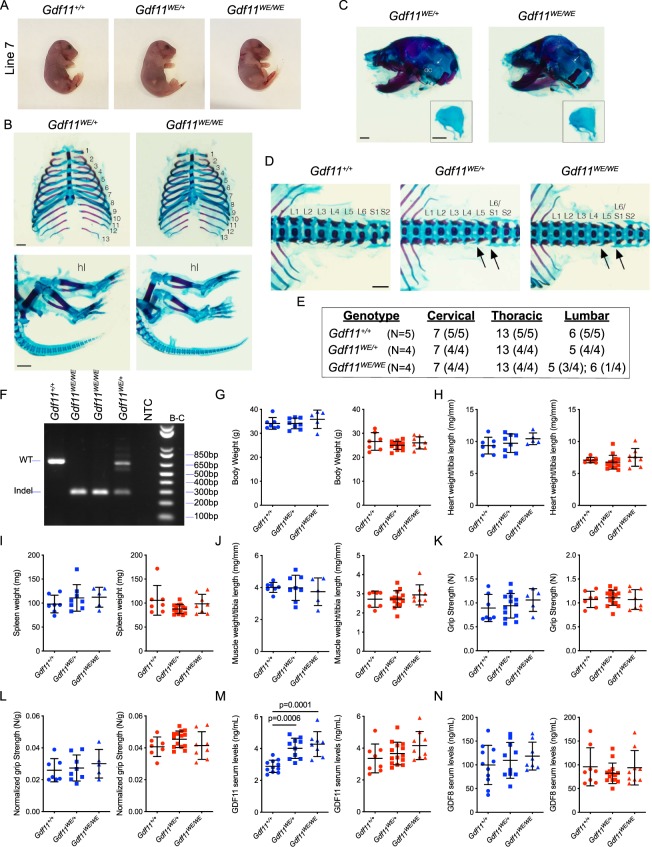
Mice homozygous for *Gdf11* deletion allele 7 (*Gdf11*^*WE*^) lack skeletal phenotypes seen in *Gdf11* loss-of-function mutants and are viable with no substantial alterations in body parameters. (**A**) Images of E18.5 embryos from line 7. *Gdf11*^*+/+*^, *Gdf11*^*WE/+*^ and *Gdf11*^*WE/WE*^ embryos all exhibit an external tail. (**B**) Skeletal analysis of *Gdf11*^*WE*/*+*^ and *Gdf11*^*WE/WE*^ embryos stained with Alcian Blue (to mark cartilage) and Alizarin Red (to mark bone). Numbers indicate thoracic vertebrae. hl: hindlimb. Scale bar: 1 mm. (**C**) Skeletal preparations of skulls from *Gdf11*^WE/+^ and *Gdf11*^WE/WE^ embryos stained with Alcian Blue and Alizarin Red. oc: otic capsule. White arrow denotes oc. Inset depicts isolated oc. Scale bar: 1 mm. (**D**) Representative images of lumbar vertebrae within *Gdf11*^*+/+*^, *Gdf11*^*WE/+*^ and *Gdf11*^*WE/WE*^ embryos. Arrows denote L5 and L6/S1 vertebrae. (**E**) Quantification of the number of cervical, thoracic and lumbar vertebrae among the genotypes indicated. (**F**) PCR analysis of *Gdf11* locus in *Gdf11*^+/+^, *Gdf11*^*WE/+*^ and *Gdf11*^*WE/WE*^ weanlings using primer pair B-C. NTC: no template control. Gel image was cropped to focus on the amplicons. Full length gel is presented in Supplementary Fig. 12D. (**G**–**J**) Quantification of (**G**), Body weight, (**H**), Normalized heart weight (relative to tibia length), **I**, Raw spleen weight, and (**J**), Normalized muscle weight (relative to tibia length) in 5–8 month old mice. N = 5–8 males (blue) and N = 7–14 females (red) per genotype. (**K,L**) Quantification of (**K**), raw and (**L**), normalized grip strength measurements (relative to body weight) in 5–8 month old mice. N = 5–8 males (blue) and N = 7–14 females (red) per genotype. Each data point represents the average of two technical replicates. (**M,N**) Quantification of (**M**) GDF11 serum levels and **N**, GDF8 serum levels in 6–8-week old mice. N = 8–11 males (blue) and N = 8–14 females (red) per genotype. Individual data points overlaid with mean ± SD.

In contrast to the other deletion alleles generated in this study, and to *Gdf11* null mice, homozygous progeny from line 7 were viable and survived through adulthood, suggesting that the *Gdf11* mutation in this line may preserve GDF11 function. *In silico* translation of this *Gdf11* gene variant is predicted to add a tryptophan (W) and a glutamic acid (E) to the C-terminus of GDF11; thus, we named this variant GDF11-WE. We further investigated the functionality of GDF11-WE by examining skeletal preparations at embryonic day 18.5 (E18.5) from GDF11-WE intercrosses. In contrast to the *Gdf11* mutants from lines 4 A, 4B, and 11 (Fig. 4C–E), and the previously reported *Gdf11* null mouse^26^, heterozygous and homozygous *Gdf11*^*WE*^ embryos did not exhibit common phenotypes observed in *Gdf11* knockout mice. For example, *Gdf11*^*WE*/*+*^ and *Gdf11*^*WE*/*WE*^ embryos exhibited a normal number of ribs (Fig. 5B), a developed otic capsule (Fig. 5C), and had a tail (Fig. 5A,B). However, upon further analysis of the vertebrae, we observed one fewer lumbar vertebra in all heterozygous embryos and most homozygous embryos analyzed (Fig. 5D,E), resulting in a predominant C7/T13/L5 vertebral pattern that is not seen in *Gdf11*^+/−^ or *Gdf11*^−/−^ mice^26^. Although the number of lumbar vertebrae can vary by strain^55–57^, this pattern is also notably different from the predominant pattern in the *Gdf11*^*WE*^ background strain– C57BL/6J–which primarily exhibits 6 lumbar vertebral segments (Fig. 5E). Altogether, these data strongly indicate that the deletion 7 allele codes for a new variant of GDF11, GDF11-WE, that maintains largely normal function during development.

We next investigated whether adult mice harboring one or two alleles encoding GDF11-WE showed any differences in circulating levels of GDF11 or GDF8. Interestingly, 5–8-week old *Gdf11*^*WE*/*+*^ and *Gdf11*^*WE*/*WE*^ males, but not females, exhibited statistically significant increases in circulating GDF11 levels relative to age-matched *Gdf11*^+/+^ controls (Fig. 5M). GDF8 levels, in contrast, were not significantly different in either males or females (Fig. 5N). These results suggest that there may be changes in ligand production and/or stability in mice possessing the GDF11-WE variant.

To assess the signaling capacity of GDF11-WE, we generated plasmid constructs enabling expression of either the mature GDF11-WE or wild-type GDF11 ligand, and tested the activity of these recombinant proteins in a well-established HEK293 SMAD3-responsive ((CAGA)_12_-promoter) luciferase assay^58–63^. In this assay, exogenous addition or co-transfection of TGF family ligands such as GDF11, together with the processing enzymes furin and tolloid-like 1, activates the SMAD2/3 signaling pathway, resulting in an increase in luciferase activity^58–63^. We transfected reporter cells with increasing amounts of plasmid encoding wild-type GDF11 or GDF11-WE (Fig. S6A,B). Transfection with GDF11-WE activated the SMAD3 reporter, but the activity was significantly less compared to cells transfected with an equal amount of plasmid encoding wild-type GDF11 (Fig. S6E). Because C-terminal modification or extension of TGF-β has been shown to negatively impact ligand dimerization and activity^64,65^, we wondered if GDF11-WE might dimerize ineffectively. We evaluated this possibility first by Western analysis and found that, whereas WT recombinant GDF11 protein is detected only as a dimer under non-reducing conditions, conditioned media from cells transfected with the GDF11-WE variant produced both the dimer and monomer signal (Fig. S6F, left). Under reducing conditions, the monomer but not the dimer, was detected for both WT recombinant GDF11 protein and GDF11-WE conditioned media (Fig. S6F, right). These data suggest that dimerization may be disrupted in the GDF11-WE variant. We further explored this idea by mutating GDF11 cysteine 73 in the mature ligand, the residue responsible for the intermolecular disulfide bond^58,66^, to a serine in both wild-type GDF11 (GDF11 C73S) and GDF11-WE (GDF11-WE C73S) (Fig. S6C,D), in order to test whether the activity of GDF11-WE, like wild-type GDF11, requires the intermolecular disulfide bond. Ligand activity was assessed using the same transfection assay described above. As expected, transfection with GDF11 C73S resulted in little to no detectable luciferase activity. Transfection with GDF11-WE C73S similarly showed little to no luciferase activity, suggesting that dimerization is still required for GDF11-WE activity (Fig. S6E). Thus, the diminished activity of the variant GDF11-WE protein relative to wild-type GDF11 may be explained by its reduced efficiency of dimerization, although alternative mechanisms related to specific effects of the added C-terminal tryptophan and glutamic acid on ligand activity cannot be excluded.

Although the activity of GDF11-WE is clearly sufficient to support largely normal development and survival after birth (Fig. 5A–F), we investigated whether adult mice harboring the GDF11-WE variant might exhibit postnatal phenotypes due to the altered levels and/or signaling activity of this variant protein. As *Gdf11* expression is upregulated is discrete immune cell lineages (Fig. 3A,B) and GDF11 functions in adulthood have previously been implicated in cardiac hypertrophy^37,67–69^, erythropoiesis^70,71^, and skeletal muscle biology^39,40^, we focused our analyses on the heart, skeletal muscle, and immune organs. We did not detect significant differences in body weight (Fig. 5G), normalized heart weight (Fig. 5H), spleen weight (Fig. 5I) or normalized muscle mass (Fig. 5J) when comparing 5–8 month-old male or female *Gdf11*^+/+^*, Gdf11*^*WE*/*+*^, and *Gdf11*^*WE*/*WE*^ mice. Furthermore, no significant differences in raw (Fig. 5K) or normalized grip strength (Fig. 5L) were observed among mice of these different genotypes. Collectively, these analyses detected no major physiological or functional defects in adult mice harboring the GDF11-WE variant (Fig. 5A–L), suggesting that the diminished activity of the GDF11-WE protein (Fig. S6E) may be compensated *in vivo* by its increased levels in males (Fig. 5M) or by other, as yet undefined, functional changes that could result from addition of the C-terminal tryptophan and glutamic acid residues.

### Absence of overt immunological phenotypes in mice harboring *Gdf11* loss-of-function or *Gdf11*^*WE*^ alleles

Given that *Gdf11* expression is upregulated is immune cell lineages (Fig. 3A,B), we examined whether mice containing *Gdf11* deletion alleles might exhibit any overt immunological phenotypes. We did not observe any obvious changes in the abundance or composition of circulating blood leukocytes in mice containing the *Gdf11* deletion alleles (Fig. S7A–G). Profiling of immune cell activation markers within T and B cell subsets similarly revealed no differences in mice harboring *Gdf11* deletion alleles (Fig. S8–10). In addition, the vast majority of circulating immune and inflammatory cytokines did not differ between *Gdf11*^+/+^ and *Gdf11*^*indel*/*+*^ mice (pooled from lines 4A, 4B, and 11), although IL-2 levels were significantly decreased (~6-fold) and M-CSF levels significantly increased (~2-fold) in *Gdf11*^*indel*/*+*^ mice relative to *Gdf11*^+/+^ mice (Table S1). No differences in serum cytokine levels were detected among age-matched *Gdf11*^+/+^, *Gdf11*^*WE*/*+*^, and *Gdf11*^*WE*/*WE*^ mice (Table S1). Mice with *Gdf11* deletion alleles also did not exhibit differences in the levels of immunoglobulin sub-classes or Ig light chain types (Table S2). Finally, we compared the relative sizes of immune organs, including the spleen, thymus, and inguinal lymph node, for cohorts of adult mice with various *Gdf11* deletion alleles. Mice heterozygous for *Gdf11*^*indel*^ alleles from lines 4A, 4B, and 11 exhibited reduced spleen weight compared to *Gdf11*^+/+^ mice (Fig. S11A); however, we did not detect significant differences in spleen weight within the *Gdf11*^*WE*^ cohort or in thymus or lymph node weights among mice in either cohort (Fig. S11A–C). Taken together, our analyses do not suggest substantial differences in homeostatic immune parameters in mice containing *Gdf11* deletion alleles, a conclusion that is further supported by two recent studies^72,73^ in which lineage-specific ablation of *Gdf11* in the hematopoietic system failed to produce overt hematoimmune phenotypes.

## Discussion

CRISPR/Cas9 gene editing tools have substantially accelerated the pace at which researchers can generate gene-modified animal models; however, reports of off-target editing events and complex genomic rearrangements following CRISPR-mediated gene editing emphasize the importance of properly validating CRISPR-modified alleles prior to phenotypic analysis. In this report, we present a series of observations stemming from our production of a knock-in *Gdf11-*IRES-GFP reporter mouse using zygotic CRISPR injections. While we succeeded in generating multiple founder animals harboring correctly targeted *Gdf11*-IRES-GFP alleles, we also generated additional alleles containing mistargeting events that produced a dramatically altered pattern of GFP expression among hematopoietic lineage cells. We further recovered multiple site-specific deletion alleles that disrupted the endogenous *Gdf11* stop codon and induced partial translation of the 3′UTR. While most of these deletions abolished GDF11 function, we also unexpectedly produced a variant allele that codes for a GDF11 protein containing two additional amino acids at its C-terminus (termed “GDF11-WE”) and retains substantial activity *in vitro* and *in vivo*. Altogether, the variety of unintended genetic alterations generated as a “side effect” of our targeted genome engineering project emphasizes both the power of CRISPR/Cas9 gene editing tools to provide important and even unexpected biological insights into gene expression and function and the absolute necessity for researchers to carefully validate proper integration of knock-in alleles. Such validation is particularly crucial when generating fluorescent reporter alleles, as additional–and in some cases difficult to detect–integration events at the target site can drastically alter phenotypic readouts, as demonstrated here.

Our results also provide new insights into the regulation of *Gdf11* expression during aging. Previous work from our group reported enriched *Gdf11* expression in the spleen of young mice and found that splenic *Gdf11* mRNA levels decline during aging^37,38^. Here, using our validated *Gdf11*-IRES-GFP reporter mouse, we extend these initial observations to track the dynamics of *Gdf11* expression with single cell resolution in peripheral blood cells of aging mice. Our findings that *Gdf11*-IRES-GFP expression is enriched within T and B lymphocytes during youth (Fig. 3A,B) suggest that lymphoid cells may represent a cellular source that contributes to the total pool of GDF11 in circulation and in tissues. In the T cell compartment, the frequency of *Gdf11*-expressing cells and the mean fluorescence intensity of *Gdf11*-IRES-GFP in the periphery do not change significantly during aging. Thus, as the overall frequency of T cells in the periphery declines during aging (Fig. 3H), the contribution to GDF11 production from T cells in the periphery likely declines with increasing age. Our findings from B cells in the periphery are more complex. While the mean fluorescence intensity of *Gdf11*-IRES-GFP in B cells increases with age, the proportion of *Gdf11*-expressing peripheral B cells increases only up to 4 months of age, and then gradually decreases. As the overall frequency of peripheral blood B cells increases during aging in mice (Fig. 3H) and the total output of *Gdf11* mRNA by B cells decreases (Fig. 3C), these results suggest that the relative abundance of B cells that do not express *Gdf11* increases with age, with partial compensation from those *Gdf11*-expressing B cells that remain. Overall, in comparison to T cells, B cells likely become more significant contributors to the total pool of GDF11 as age increases. Moving forward, the validated *Gdf11*-IRES-GFP reporter mouse described here will provide a useful tool to further interrogate *Gdf11* expression dynamics and the regulation of *Gdf11*-expressing cells in multiple physiological contexts, including development, homeostasis, aging, and disease.

Our findings that *Gdf11*-IRES-GFP expression in validated reporter mice is enriched in lymphoid cells are consistent with published data sets assessing *Gdf11* expression levels^50–52^. A peculiar finding from our analysis of mice harboring alternative *Gdf11*-IRES-GFP integration events, which remains unresolved, is why the lymphoid-biased GFP expression pattern observed in the correctly targeted reporter lines switched to a myeloid-biased GFP expression pattern in the incorrectly targeted reporter lines. One possible explanation is that the integration of sequences from the plasmid backbone at the target site in the incorrectly targeted lines structurally alters a regulatory element that controls gene expression selectively within either the lymphoid or myeloid lineage. The striking differences in the GFP expression pattern between the different alleles suggest that DNA-based elements may exist that have the capacity to switch gene expression patterns from the lymphoid to the myeloid lineage, which may be interesting to dissect in future studies.

In addition to the intended generation of a *Gdf11*-IRES-GFP knock-in allele, zygotic targeting at the *Gdf11* locus also produced several *Gdf11* indel variants, which revealed unexpected insights into how modifications at its C-terminus can alter GDF11’s biological function. Previous attempts to add epitope tags to the C-terminus of TGF-β molecules have disrupted protein processing and significantly diminished ligand bioactivity^64,65^. Thus, we expected that adding exogenous amino acids to the GDF11 C-terminus would similarly impair its function. Consistent with this notion, the deletions detected in lines 4A, 4B, and 11, which add 156, 14 and 32 additional amino acids, respectively, to the C-terminus of the protein, each exhibited skeletal and renal phenotypes consistent with *Gdf11* null phenotypes^26,33^ (Figs. 4C,D and S5A,B). These results suggest that extension of the GDF11 mature ligand by as few as 14 amino acids renders the protein non-functional. On the other hand, the GDF11-WE variant, which is predicted to extend the GDF11 mature ligand by only two amino acids, does not recapitulate GDF11 loss-of-function phenotypes *in vivo*, as mice homozygous for this allelic variant showed no tail malformations or ectopic ribs (Fig. 5A,B) and survive to adulthood with no major physiological defects. Interestingly, we did detect a decrease in the number of lumbar vertebrae in all heterozygous embryos and a majority of homozygous *Gdf11*^*WE*^ embryos analyzed (Fig. 5D,E). While this feature has been reported in other mutant animals^74–77^, it has not been reported in mice harboring *Gdf11* null alleles^26,33^. Indeed, the loss of lumbar vertebra seen in *Gdf11*^*WE*/*+*^ and *Gdf11*^*WE*/*WE*^ mice appears opposite to that of *Gdf11*^+/−^ and *Gdf11*^−/−^ mice, which show expanded numbers of lumbar (and thoracic) vertebrae^26,33^, making it unlikely that this variant allele produces a loss-of-function phenotype. Studies investigating skeletal phenotypes in mouse models lacking *Fst* and/or *Gasp2*, known inhibitors of GDF11, have similarly reported five lumbar vertebrae^34,78^, along with other skeletal abnormalities. Further investigation will be necessary to determine if and how the addition of a tryptophan and glutamic acid to the C-terminus of GDF11 may alter its biological functions and to tease apart whether the specific addition of these two amino acids, as opposed to any others, is key to the preservation of many of GDF11’s normal *in vivo* functions by this variant ligand. Another interesting finding emerging from this study is the presence of a discernable hole within the otic capsule of E18.5 embryos containing one or two copies of a *Gdf11* null allele^26^, as well as in several of the *Gdf11* indel embryos (Fig. 4E). While the mechanism by which this hole forms, or is retained, during development in the absence of two functional copies of *Gdf11* remains unclear, it would be interesting to investigate whether this phenotype has any biological effects.

Finally, in addition to the novel insights into the regulation and function of GDF11, our study has broad implications for utilizing CRISPR/Cas9 genome editing approaches to generate precise gene-modified animal models via HDR. From our efforts to generate a knock-in GFP reporter mouse by CRISPR/Cas9, we generated alleles that were targeted to the intended genomic locus while also containing an additional on-target integration event that strikingly disrupted reporter gene expression. These findings are consistent with a prepublication report from another group that also detected head-to-tail insertions of donor templates at the target site following zygotic CRISPR/Cas9 injections in mice^79^. Disturbingly, these additional integration events were not detected by PCR analysis, emphasizing the need to perform additional screening methods (e.g. Southern blotting, TLA sequencing) to confirm appropriate targeting. For exploratory and pre-clinical CRISPR/Cas9-mediated HDR studies, we recommend verifying correct CRISPR targeting events using these additional methods, as incorrect on-target events can occur in a significant percentage of founder animals and can substantially alter phenotypic readouts.

## Materials and Methods

### pCas9 and sgRNA expression plasmids

The pCas9-mCherry plasmid was generated by replacing the GFP sequence from the pCas9-GFP plasmid with the mCherry sequence. pCas9-GFP was a gift from Kiran Musunuru (Addgene plasmid # 44719; http://n2t.net/addgene:44719; RRID: Addgene_44719)^80^.

Four different sgRNAs were designed to target exon 3 of *Gdf11* immediately after the stop codon and at the beginning of the 3′UTR. sgRNA sequences were designed using the online tool http://www.genome-engineering.org, and the sgRNA target sequences are listed in Table S3. For each sgRNA, a pair of oligos were designed containing BbsI overhangs and a G nucleotide to facilitate U6-driven expression. Oligo nucleotides were annealed, digested with BbsI (New England Biolabs) and ligated into a BbsI-digested plasmid backbone using T4 ligase (New England Biolabs). The backbone was generated by removing the GFP sequence from the gRNA_GFP_T1 plasmid to contain 2 BbsI sites. gRNA_GFP-T1 was a gift from George Church (Addgene plasmid # 41819; http://n2t.net/addgene:41819; RRID Addgene_41819)^9^. The plasmid was transformed into Top10 cells and confirmed by Sanger sequencing. Plasmids were purified using the EndoFree Plasmid Maxi Kit (Qiagen).

### *Gdf11*-IRES-GFP Homology-Directed Repair (HDR) plasmid construction and purification

The left homology arm, IRES-GFP, right homology arm and pUC19 backbone fragments were cloned using Gibson Assembly Master Mix (New England Biolabs), transformed into TOP10 cells (Thermo Fisher) and validated by Sanger sequencing. For transfection experiments, the HDR plasmid was purified using the EndoFree Plasmid Maxi Kit (Qiagen). For zygote injections, the HDR plasmid was further purified with phenol-chloroform three times, chloroform twice and ethanol precipitated. Plasmid DNA was resuspended in TE buffer, treated with the Ultraclean Endotoxin Removal Kit (MO-bio), ethanol precipitated, rinsed 5 times with 70% ethanol and re-suspended in nuclease-free water.

### Cell culture and transfections

C2C12 cells were cultured in DMEM (Thermo Fisher) containing 10% fetal bovine serum (Atlanta Biologicals) and 1X penicillin/streptomycin (Thermo Fisher) at 37 °C in 5% CO_2_. Immediately prior to transfection, C2C12 cells were transferred to fresh media (DMEM, 10% fetal bovine serum) lacking 1X penicillin/streptomycin. To test the on-target cutting efficiencies of the sgRNAs, 50,000 cells were transfected with 250 ng of Cas9-2A-mCherry plasmid and 250 ng of the sgRNA expression plasmid. For Cas9-only transfections, 50,000 cells were transfected with 250 ng of Cas9-2A-mCherry plasmid and 250 ng of TR30022 plasmid as a control (Origene). Plasmids were transfected with Lipofectamine 3000 (Thermo Fisher) according to manufacturer’s instructions. Cells were harvested 3 days post transfection for analysis. To test targeting of the *Gdf11*-IRES-GFP HDR template to the *Gdf11* locus, 50,000 cells were transfected with 125 ng of Cas9-2A-mCherry plasmid, 125 ng of sgRNA expression plasmid and 250 ng of the *Gdf11*-IRES-GFP HDR template using Lipofectamine 3000 (Thermo Fisher) according to manufacturer’s instructions. Transfected cells were imaged 4 days post transfection by fluorescence microscopy. For analysis of HDR at the *Gdf11* locus by PCR, cells were harvested 3 days post transfection for FACS purification.

### Flow cytometry and fluorescence activated cell sorting (FACS)

Transfected C2C12 cells were detached with Accutase (Innovative Cell Technologies) and collected in staining media (Hank’s Balanced Salt Solution (HBSS) (Thermo Fisher); 2% fetal bovine serum (Atlanta Biologicals) and 10 mM ETDA). Cells were stained with 7-AAD viability dye (BD Biosciences, 559925, 1:20 dilution). Live, Cas9-2A-mCherry^+^ cells were sorted into staining media and resuspended in DNA quick extract (Epicentre). For FACS isolation of GFP^high^ and GFP^low^ splenocytes from *Gdf11*-IRES-GFP mice, spleens were harvested and passed through a 40μm filter using a syringe plunger. Red blood cells were lysed using ACK lysis buffer (Thermo Fisher) and cells were resuspended in staining media. Splenocytes were stained with 7-AAD viability dye immediately prior to FACS and live GFP^high^ and GFP^low^ splenocytes were sorted into staining media, pelleted and resuspended in Trizol (Thermo Fisher). Cell sorting was performed on a BD FACS Aria II at the Harvard Stem Cell and Regenerative Biology Flow Cytometry Core.

To evaluate GFP fluorescence in peripheral blood cell cells from *Gdf11*-IRES-GFP mice by flow cytometry, cells were incubated with purified rat anti-mouse CD16/32 (Mouse BD Fc Block, 2.4G2, BD Biosciences, 1:50 dilution) for 5 minutes on ice. Cells were subsequently stained with the following antibodies for 30 minutes on ice: anti-CD3-PECy7 (17A2, Biolegend, 100220, 1:65 dilution), anti-CD19-PE (eBio1D3, Thermo Fisher, 12-0193-82, 1:100 dilution), anti-CD11b-APC (M1/70, Biolegend, 101212, 1:200 dilution) and anti-Ly-6G-Pacific Blue (1A8, Biolegend, 127612, 1:200 diution). 7-AAD was used as a viability dye (BD Biosciences, 559925, 1:20 dilution). Flow cytometry analysis was performed on a BD LSR II at the Harvard Stem Cell and Regenerative Biology Flow Cytometry Core. To normalize *Gdf11*-IRES-GFP fluorescence intensity levels over time, BD CaliBRITE FITC beads (BD biosciences) were used to standardize 488 nm laser alignment and intensity at each analysis time point.

To profile immune cell activation markers and T and B cell subsets in peripheral blood from mice containing *Gdf11* deletion alleles, ACK-lysed peripheral blood cells were incubated with rat anti-mouse CD16/32 (Mouse BD Fc Block, 2.4G2, BD Biosciences, 1:50 dilution) for 5 minutes on ice. One half of the sample was incubated with the following antibodies for 30 minutes on ice to evaluate T cells: anti-CD3-PECy7 (17A2, Biolegend, 100220, 1:65 dilution), anti-CD4-PerCPCy5.5 (RM4-5, BD Biosciences, 561115, 1:200 dilution), anti-CD8-PE (53-6.7, Biolegend, 100707,1:200 dilution), anti-CD44-APC (IM7, Biolegend, 103011, 1:200 dilution), anti-CD62L-eFluor450 (MEL-14, Thermo Fisher, 1:200 dilution), anti-CD25-FITC (3C7, Biolegend, 101908, 1:25 dilution) anti-CD152 (CTLA-4)-PE-Dazzle 594 (UC10-4B9, Biolegend, 106317, 1:75 dilution), and anti-CD279 (PD-1)-APCCy7 (29F.1A12, Biolegend, 135225, 1:100 dilution). The second half of the sample was incubated with the following antibodies for 30 minutes on ice to evaluate B cells: anti-CD19-PE-CF594 (1D3, BD Biosciences, 562291, 1:200 dilution), anti-B220-FITC (RA3-6B2, Thermo Fisher, 11-0452-85, 1:100 dilution), anti-CD80-PerCPCy5.5 (16-10A1, BD Biosciences, 560526, 1:50 diltuion), anti-CD86-PECy7 (GL-1, Biolegend, 105013, 1:100 dilution), and anti-IgM-PE (eB121-15F9, Thermo Fisher, 12-5890-81, 1:200 dilution). Zombie Aqua was used as a viability dye (Biolegend, 423102, 1:300 dilution). Data were collected on a BD LSR II at the Harvard Stem Cell and Regenerative Biology Flow Cytometry Core and analyzed using FlowJo version 10.5.3 (TreeStar).

### Magnetic activated cell sorting of CD19+ and CD19− cells

Spleens were dissociated into single cell suspensions using the gentleMACS dissociator in manufacturer provided C-Tubes containing 3 mL MACS buffer (PBS, pH 7.2, 0.5% BSA, 2 mM EDTA). This suspension was passed through 40 μM filters into 15 ml Falcon tubes and placed on ice. Cells were counted using a hemocytometer, resuspended to a density of 10^8^ cells/mL, and incubated with a proportional volume of MACS CD19 microbeads (10 μL beads/10^7^cells) at 4 °C for 15 minutes. Using the MACS Separator under a positive selection program, CD19+ cells bound to microbeads were held to a magnetic column while CD19- cells flowed through. Each sample was double sorted using this selection method. CD19+ and CD19− fractions were counted using a hemocytometer with Trypan blue to determine the proportion of live CD19+ cells separated from whole spleen. Approximately 4,000 cells from the CD19+ and CD19− fractions were assessed by flow cytometry to determine purity of fractions and efficacy of magnetic separation. Cells were resuspended in Trizol and stored at −80 °C.

### T7 Endonuclease 1 (T7E1) mismatch detection assay

Genomic DNA was extracted from FACS-purified mCherry^+^ C2C12 cells with 30 μL of DNA Quick Extract Buffer (Epicentre). The *Gdf11* target region was PCR amplified (see Table S4 for primer sequences) using Q5 polymerase (New England Biolabs) according to the manufacturer’s protocol. PCR products were purified using a PCR purification kit (Qiagen), denatured at 95 °C for 5 minutes and re-annealed from 95 °C-85 °C at a rate of −2 °C/second, and from 85 °C-25 °C at a rate of -0.1 °C/second to form heteroduplexes. Reannealed heteroduplexes were incubated with 25 units of T7E1 enzyme (New England Biolabs) at 37 °C for 15 minutes, and products from mismatch assay were visualized on a 2% agarose gel.

### *In vitro* transcription

The T7 promoter was added to the sgRNA by PCR amplification of the sgRNA plasmid template using the primers listed in Table S4. The T7-sgRNA3 amplicon was gel purified and used as the template for *in vitro* transcription using the MEGAscript T7 kit (Life Technologies). The *in vitro* transcribed sgRNA3 was purified using the MEGAclear Transcription Clean-Up Kit (Thermo Fisher) and eluted in nuclease-free water. The sgRNA was ethanol precipitated with ammonium acetate, rinsed 5 times with 70% ethanol and re-suspended in nuclease-free water.

### Zygote injections

Zygote injections were performed by the Genome Modification Facility at Harvard University using a standard protocol as described previously^14^. Briefly, superovulated C57BL/6J females were mated to C57BL/6J males and fertilized zygotes were harvested from oviducts the following day. Zygotic pronuclei were injected with a 5 μL mixture of (a) 1 μL purified HDR template (200 ng/μL), (b) 0.5 μL *in vitro* transcribed sgRNA (50 ng/μL), (c) 1 μL of either transfection-ready Cas9 SmartNuclease mRNA (eukaryotic version) (System Bioscience) (100 ng/μL) or 1 μL Cas9 protein with NLS (injection ready) (PNA Bio) (100 ng/μL) and (d) 2.5 μL of nuclease-free water. Injected zygotes were transferred into the oviducts of pseudopregnant ICR females (CD-1; Charles River Laboratories) at 0.5 days post coitum.

### Evaluation of CRISPR-mediated HDR at *Gdf11* locus

For analysis of *Gdf11*-IRES-GFP targeting in C2C12 cells and mice, genomic DNA was extracted using DNA Quick Extract Buffer (Epicentre) and PCRs were performed with Q5 polymerase (New England Biolabs) according to the manufacturer's protocols. The targeted region of the *Gdf11* locus was PCR amplified by semi-nested PCR (see Table S4 for primers used).

F_0_ founders were first screened for the presence of the *Gdf11*-IRES-GFP using an internal PCR amplicon, which is the same amplicon used for the T7E1 assay (see Table S4). Of those mice containing the *Gdf11*-IRES-GFP sequence, the F_0_ founders were subsequently screened using two different amplicons that spanned the junction between the genomic DNA and homology arms (see Table S4 for PCR primer sequences).

### Southern blot

Genomic DNA was isolated from mouse tail in DNA digestion buffer (10 mM Tris pH 8.0, 5 mM EDTA, 0.1 M NaCl, 1% SDS) containing 100 μg proteinase K overnight at 56 °C followed by phenol-chloroform extraction, ethanol precipitation and re-suspension in TE buffer. Genomic DNA was digested overnight with 100 units of NcoI-HF (New England Biolabs) at 37 °C, separated on a 0.8% agarose gel and transferred to a positively charged nylon membrane (Roche). Membranes were hybridized with digoxigenin (DIG)-labelled internal probes (Roche) and visualized by chemiluminescence using an AP-anti-DIG antibody (Roche)^81^ and CDP-Star (Roche). Membranes were stripped using 0.2 M sodium hydroxide with 1% SDS at 37 °C, rehybridized with DIG-labelled external probes and visualized using the same chemiluminescence protocol. DIG-labelled probes were generated by PCR using the DIG probe synthesis kit (Roche) according to manufacturer’s instructions (see Table S4 for primer sequences).

### Targeted locus amplification (TLA) sequencing

Spleens from *Gdf11*-IRES-GFP mice were homogenized and subjected to ACK lysis. Splenocytes were frozen and shipped to Cergentis (Utrecht, the Netherlands) for TLA sequencing analysis as described previously^48,49^. Briefly, splenocyte genomic DNA was crosslinked, digested and re-ligated. Genomic DNA was subsequently purified and circular TLA templates were amplified using two independent sets of inverse primers complementary to the transgene (see Table S4 for primer sequences). Following amplification of the targeted locus, PCR amplicons were purified and sequencing libraries were prepared for Illumina sequencing.

### RNA isolation, cDNA synthesis and real-time PCR

Total RNA was isolated using Trizol (Thermo Fisher) according to the manufacturer’s protocol. RNA concentration was determined using a NanoDrop (Thermo Fisher) and equal amounts of RNA were added to cDNA synthesis reactions. cDNA was synthesized using the SuperScript III First-Strand Synthesis SuperMix for qRT-PCR (Thermo Fisher) according to the manufacturer’s protocol. Real-time PCR was performed on an ABI Prism 7900HT sequence detection system using RT^2^ SYBR Green/ROX FAST master mix (Qiagen) using the primers listed in Table S4. Relative quantification was calculated as 2(^−ΔΔCT^) using *β-actin* as a reference gene. For analysis of *Gdf11* expression from the MACS-purified CD19+ and CD19- splenic cells, real-time PCR analysis was performed with Taqman probes for *Gdf11* (Mm01159973_m1, Taqman Gene Expression Assays, Thermo Fisher) and *Hprt* (Mm01545399_m1, Taqman Gene Expression Assays, Thermo Fisher). Relative quantification was calculated as 2(^−ΔΔCT^) using *Hprt* as a reference gene.

### *in situ* Hybridization

Antisense DIG-labeled riboprobes against *Gdf11 and Gfp* were *in vitro* transcribed from PCR fragments (Roche, 1175025910) that were generated using primers listed in Table S4. PCR products were sequenced verified prior to *in vitro* transcription. *In situ* hybridization was performed on four embryos per genotype at E10.5. For whole-mount *in situ* hybridization, embryos were fixed in 4% paraformaldehyde for 18 hours at 4 °C, washed 3x for 10 minutes in 1X PBS, and then dehydrated through a graded series of 25%, 50%, 75% methanol/0.85% NaCl incubations. Embryos were stored in 100% methanol at −20 °C before *in situ* hybridization. Embryos were rehydrated through a graded series of 75%, 50%, 25%, methanol/0.85% NaCl incubations and finally washed in 2x PBS with 0.1% tween-20 (PBST). E10.5 embryos were treated with 10 mg/mL proteinase K in PBST for 30 minutes at room temperature. Samples were then fixed in 4% paraformaldehyde/0.2% glutaraldehyde in PBST for 20 minutes at room temperature and washed 2x in PBST. Embryos were incubated in pre-hybridization solution for 1 hour at 68 °C, and then incubated in 500 ng/mL of riboprobe at 68 °C for 16 hours. Post-hybridization, samples were washed in stringency washes and incubated in 100 μg/mL RNase A at 37 °C for 1 hour. Embryos were washed in 1X maleic acid buffer with 0.1% tween-20 (MBST) and then incubated in Blocking Reagent (Roche) with 10% heat inactivated sheep serum (Sigma # S2263) for 4 hours at room temperature. Anti-DIG antibody (Roche, 11093274910) was used at 1:5000 and samples were incubated for 18 hours at 4 °C. Samples were washed 8x with MBST for 15 minutes at room temperature, 5x in MBST for 1 hour at room temperature, and then 1x in MBST for 16 hours at 4 °C. Prior to development, embryos were incubated 3x in NTMT (100 mM NaCl, 100 mM Tris-HCl (pH9.5), 50 mM MgCl2, 0.1% tween-20, and 2 mM levamisole), and the *in situ* hybridization signal was developed by adding BM Purple (Roche, 11442074001) for 5 hours at room temperature. After the colorimetric development, samples were fixed in 4% paraformaldehyde and cleared through a graded series of glycerol / 1X PBS and stored in 80% glycerol.

### Animals

All animal housing, handling and experiments were approved by the Institutional Animal Care and Use Committee at Harvard University/Faculty of Arts and Sciences and carried out in accordance with the relevant guidelines and regulations. For timed breedings, the presence of a vaginal plug indicated an embryonic day 0.5 (E0.5) timepoint.

### Skeletal preparation protocol

E17.5 and E18.5 embryos were dissected, deskinned, eviscerated, fixed in 100% ethanol for 24 hours at room temperature and dehydrated in 100% acetone for 24 hours at room temperate. Skeletons were incubated in staining solution (0.3% Alcian blue 8GS (Sigma), 0.1% Alizarin red S (Sigma), 70% ethanol and 5% acetic acid) for 72 hours. Skeletons were cleared in 1% potassium hydroxide solution for 24 hours, followed by additional clearing increasing the percentage of glycerol from 20% to 50% to 80% after every 24-hour period. Embryos were imaged and stored in 80% glycerol with 1% potassium hydroxide.

### Grip strength assay

Mice were allowed to grasp the metal grid of the grip strength meter (Bioseb) with only their forelimbs. Mice were pulled backwards, and the force applied to the grid was recorded in Newtons. Raw force measurements were performed in duplicate, recorded and averaged. The force normalized to the body weight for each animal was calculated.

### Collection of peripheral blood, serum, and tissues

Peripheral blood samples were collected from adults via the lateral tail vein into PBS (Gibco) containing 10 mM EDTA and stored on ice. Red blood cells were sedimented using 2% Dextran (Sigma) in PBS for 30 minutes at 37 °C, followed by washing with Staining Media (HBSS containing 2% FBS), incubation with ACK lysis buffer (Thermo Fisher) and filtration through a 40 μM cell strainer for flow cytometry analysis.

For serum collection, peripheral blood was collected via the tail vein into Microtainer tubes with a serum separator (BD) and incubated for at least 30 minutes at room temperature. Samples were centrifuged at 2000xg for 10 minutes at room temperature. The upper serum layer was transferred to a new tube and stored at −80 °C prior to analysis.

For tissue collection from adults, mice were euthanized with CO_2_ prior to tissue collection. The heart was dissected, cleaned in PBS and dried on a paper towel prior to weighing. The spleen, thymus, and inguinal lymph node were dissected, weighed and either stored in staining media or snap-frozen in liquid nitrogen. The tibialis anterior and extensor digitorum longus (TA and EDL) muscles were harvested and weighed. The tibia bone was cleaned and measured using calipers.

For tissue collection from E18.5 embryos, pregnant females were euthanized with CO_2_ and embryos were dissected in PBS. Urogenital tracts were dissected in PBS and imaged. Embryos were scored for the presence of 0, 1 or 2 kidneys by visual inspection.

### Complete blood count (CBC) analysis

Peripheral blood was collected via tail vein in EDTA-coated Microtainer tubes (BD) and kept on ice. CBCs were analyzed on an Element HT5 (HESKA) instrument.

### Cytokine analysis

Serum was isolated and shipped to Eve Technologies for cytokine analysis using the Mouse Cytokine Array/Chemokine Array 31-Plex (MD31). To enable statistical analyses, values reported as out of range below the 4 or 5 parameter logistic standard curve were inputted as 0.02 pg/mL, the lowest extrapolated value that can be calculated by the standard curve mathematical formula.

### Immunoglobulin isotyping array

Serum was diluted 100-fold in Sample Diluent and hybridized to the Rapid Mouse Ig Isotyping Array (Ray Biotech, Norcross, GA, USA) according to the manufacturer’s protocol. Following hybridization, slides were shipped to Ray Biotech for scanning and data extraction. Normalized fluorescence values were calculated using the AAM-ISO-G1 Analyzer program (Ray Biotech, Norcross, GA, USA).

### Liquid chromatography tandem mass spectrometry of GDF11 and GDF8

A minimum of 100 μL of mouse serum was submitted for quantitative liquid chromatography tandem mass spectrometry to detect GDF11 and GDF8 protein concentrations^53^ at the Brigham and Women’s Hospital Brigham Research Assay Core (BRAC). Briefly, mouse serum was denatured, reduced and alkylated, followed by pH-based fractionation using cation ion exchange SPE; the appropriate elution fraction was digested with trypsin. After desalting and concentrating of the tryptic digest, the peptide mixture was separated and eluted by liquid chromatography followed by mass spectrometric analysis operated in positive electrospray ionization mode. The most intensive and unique proteotypic peptides from GDF11 and GDF8 as surrogate peptides along with heavy-labeled unique peptides as internal standards were used for quantitative determination of GDF11 and GDF8.

### Site-directed mutagenesis

Primers used for site-directed mutagenesis were designed to target nucleotides in our previously described chimeric DNA template that consisted of human GDF8 prodomain attached to the mature GDF11 protein sequence, annotated GDF8pro/GDF11mature in the pRK5 vector (see Table S4 for primer sequences)^58^. Successful mutagenesis for all constructs was confirmed by sequencing.

### HEK293 (CAGA)_12_ luciferase assay

As previously described, HEK293-(CAGA)_12_ luciferase reporter cells were plated at ~2 × 10^4^ cells/well and grown overnight in 96-well plates and transiently co-transfected with plasmids containing the GDF8pro/GDF11mature constructs (25–100 ng; pRK5), human tolloid-like 1 (TLL1; 50 ng; pcDNA3), and human furin (50 ng; pcDNA4) using Mirus LT-1 transfection reagent. Empty pRK5 vector was added for a total of 200 ng DNA transfected/well. Cells were transfected for 6 hours followed by removal of growth medium, replaced by serum-free medium and cultured for an additional 24 hours. Cells were lysed and luminescence was recorded immediately using a BioTek Synergy H1 Hybrid plate reader. Values from triplicates were averaged and each average was normalized to the negative control cells transfected with empty pRK5 vector, furin, and TLL1. The activity data was imported into GraphPad Prism for graphing and statistical analysis.

### Western blot

Conditioned media was collected from Expi293 cells (Invitrogen; #A14527) four days following transfection with the GDF11-WE plasmid. Increasing volumes of conditioned media (5 μL, 10 μL, or 20 μL) or 10 ng of recombinant GDF11 (rGDF11) protein (Peprotech) were processed under non-reducing or reducing conditions. For reducing conditions, samples were denatured in the presence of β-mercaptoethanol prior to separation on a 4–15% SDS gel (Bio-Rad; #4561086). For non-reducing conditions, samples were denatured, separated on an SDS gel, and the gel was reduced after separation by electrophoresis in the presence of 575 mM β-mercaptoethanol diluted in SDS running buffer (25 mM Tris pH 8.5, 192 mM glycine, 0.1% SDS) and incubated for 20 minutes at room temperature. Proteins were transferred to a PVDF membrane using the Bio-Rad Trans-Blot Turbo™ Transfer System. The membrane was blocked with 5% milk diluted in TBST (10 minutes, rocking) and incubated with primary antibody (Rabbit Anti GDF11/GDF8 Abcam Cat# EPR4567^38^) at a 1:1000 dilution in 1.5% milk in TBST (2 hours, rocking). The membrane was washed 5 times with TBST (5 minutes per wash) and incubated with secondary antibody (Anti Rabbit R&D antibody Cat#HAF008^82^) at a 1:3000 dilution in 1.5% milk in TBST (1 hour, rocking). The membrane was washed 5 times with TBST (5 minutes per wash), incubated using Pierce ECL Plus Western Blotting Substrate, and developed.

### Equipment and settings

Agarose gel electrophoresis images (Figs. 1B,D, 5F, S1C,D, S2A,B and S12A,D) were acquired on a Biorad Molecular GelDoc XR imaging system using Biorad ImageLab software. For all images other than Fig. S12A, digital images were imported into Photoshop CC 2017 (Adobe), cropped when indicated, and no other post-image processing was performed. For the image in S12A, the gel image was printed from ImageLab software onto Thermal Paper (110 mM × 20 M, Bio Doc-IT accessory, Genesee Scientific, #K65HMCE) using a Mitsubishi P95DW printer and digitally scanned using a Scanjet 8300 (HP). The image was rotated and cropped to focus on the agarose gel using Photoshop CC 2017 (Adobe), and no other post-image processing was performed.

Southern blot images (Figs. 2A,B, S3A,B and S12B,C) were acquired by exposing membranes to Biomax Maximum Sensitivity (MS) autoradiography film (Kodak). Films were fixed and developed using an M35A X-Omat Processor (Kodak) and digitally scanned using a Scanjet 8300 (HP). Brightness and contrast levels were adjusted uniformly across the entire scanned images using Photoshop CC 2017 (Adobe).

For *in situ* hybridization images (Fig. 2K,L), embryos were placed in a 10 cm^2^ tissue culture dish filled with 80% glycerol in PBS. Embryos were imaged on a Leica M216FA stereomicroscope (Leica Microsystems) equipped with a DFC300 FX digital imaging camera. Images were acquired with Leica Application Suite v2.3.4 R2 software. Forelimbs were acquired at 51x magnification and embryo images were acquired at 17x magnification. *In situ* hybridization images were processed in Adobe Bridge, where the following adjustments were applied to all images: exposure +1.10, tint +13, temperature +3.

For mouse embryo images (Figs. 4C and 5A), embryos were photographed using a Samsung Galaxy S9+ camera. Brightness and contrast levels were adjusted uniformly across the entire scanned images using Photoshop CC 2017 (Adobe). Skeletal preparations (Figs. 4D,E and 5B–D) were photographed using a Nikon D7000 with a Nikon 28–105 lens with macro. Images were captured in Nikon Electronic Format (NEF) and adjusted in Adobe Camera Raw plugin to a + 0.70 exposure and color temperature of 6700 K.

For embryonic urogenital tract images (Fig. S5A), tissues were imaged using a Leica MZ16 FA fluorescence stereomicroscope equipped with a Leica DFC7000 T camera. Images were captured in.lif format using Leica Application Suite X and exported as.tif files with no image post-processing performed.

Transfected C2C12 cells (Fig. S1F) were imaged on a Zeiss Observer.D1 inverted fluorescence microscope using AxioVision Rel 4.7 software. Western blot images (Fig. S6F) were acquired by exposing membranes to GeneMate blue autoradiography film. Films were fixed and developed using an M35A X-Omat Processor (Kodak) and digitally scanned using an Epson GT-1500. Images were flipped horizontally, converted to grayscale, and brightness levels were adjusted uniformly across the entire scanned images using Photoshop CC 2017 (Adobe).

### Statistical analysis

For the *Gdf11*-IRES-GFP mice, we performed statistical analyses to assess the association between aging and GFP+ cell frequency as well as GFP mean fluorescence intensity (MFI). For the percentage analyses, we fit the logistic regression model and applied the generalized estimating equations (GEE) approach^83,84^ using the gee package in the statistical software R (v3.5.3)^85^. The GEE estimation method allows the analysis to account for correlations between repeated measurements. For normalized MFI responses, we used linear regression models with the GEE approach. For each of the analyses, the adequacy of the statistical models was assessed through the visual inspection of standardized residuals and fitted values. Both the residual plot and time plot of the mean response in Fig. 3E,G suggested that the B cell percentage and MFI data required multiple linear trends over time. Therefore, we used a piecewise regression model, which allows two linear models to be fit to the B cell data, one for ≤4 months of age and the other for age >4 months of age. Two-sided p-values for all tests are presented in Fig. 3D–G.

For all other analyses, comparisons between three or more groups were performed by One-Way ANOVA followed by Bonferroni post-test correction. Observed differences with p < 0.05 were considered as statistically significant.

## Supplementary information

Supplementary Information

## Data Availability

For original data, please contact Amy Wagers at amy_wagers@harvard.edu.
