# Supplementary material for: Variation in zygotic CRISPR/Cas9 gene editing outcomes generates novel reporter and deletion alleles at the Gdf11 locus
Source: Sci Rep. 2019 Dec 9;9:18613. doi: 10.1038/s41598-019-54766-y (PMC6901511; doi:10.1038/s41598-019-54766-y)
Supplement: Supplementary file 1 — Supplementary Information [file 41598_2019_54766_MOESM1_ESM.docx]

**Variation in zygotic CRISPR/Cas9 gene editing outcomes generates novel reporter and deletion alleles at the *Gdf11* locus**

Jill M. Goldstein^1,2,3^, Austin Valido^1^, Jordan P. Lewandowski^1,2^, Ryan G. Walker^1,2^, Melanie J. Mills^1,2^, Kathleen A. Messemer^1,2,3^, Paul Besseling^1,2^, Kyu Ha Lee^4^, Samuel J. Wattrus^1,2^, Miook Cho^1,2,3^, Richard T. Lee^1,2^, Amy J. Wagers^1,2,3,5^

^1^Department of Stem Cell and Regenerative Biology, Harvard University, Cambridge, MA 02138, USA

^2^Harvard Stem Cell Institute, Cambridge, MA 02138, USA

^3^Paul F. Glenn Center for the Biology of Aging, Harvard Medical School, Boston, MA 02215, USA

^4^Department of Nutrition, Harvard T.H. Chan School of Public Health, Boston, MA 02115, USA

^5^Section on Islet Cell and Regenerative Biology, Joslin Diabetes Center, Boston, MA, 02215, USA

Correspondence should be addressed to:

Amy J. Wagers

amy_wagers@harvard.edu

Department of Stem Cell and Regenerative Biology

Harvard University and Harvard Stem Cell Institute

7 Divinity Avenue

Cambridge, MA 02138

**Supplementary Figures**

**
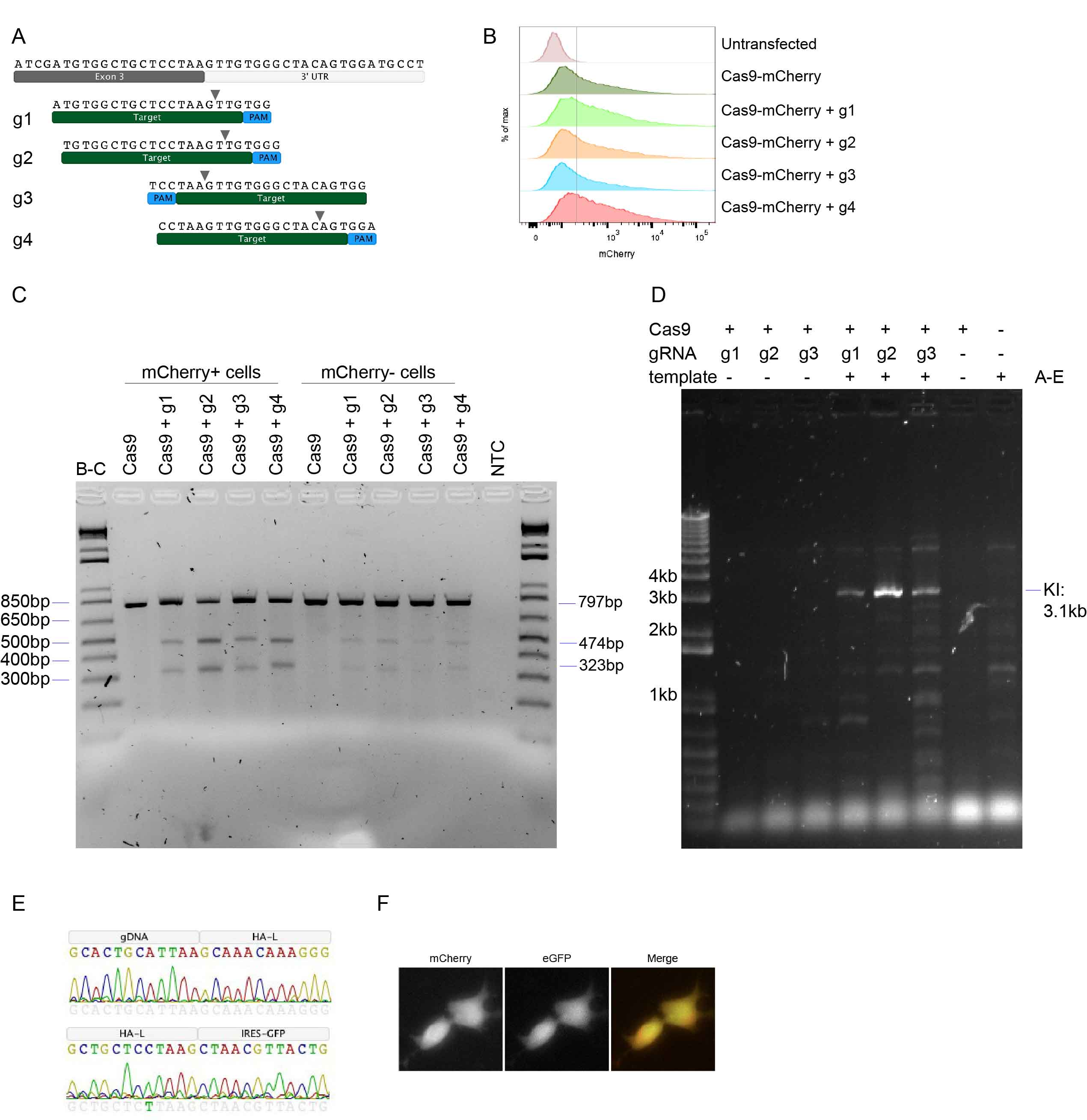
Supplementary Figure 1.** Validation of CRISPR/Cas9-mediated targeting of an IRES-GFP reporter to the *Gdf11* locus in C2C12 cells. **A**, Schematic of sgRNAs targeting *Gdf11* with the target sequence and PAM indicated. The predicted cut site for Cas9 resides approximately 3bp upstream of the PAM and is denoted by a gray triangle. **B**, Flow cytometry analysis of mCherry expression in untransfected, Cas9-mCherry only or Cas9-mCherry/sgRNA-transfected C2C12 cells. **C**, T7E1 endonuclease assay of *Gdf11* sgRNA targeting in FACS purified Cas9-mCherry+ and mCherry- cells C2C12 cells. PCR amplification using primer pair B-C produced a 797bp amplicon. Digested products were detected at 474bp and 323bp. NTC: No template control. Gel image is uncropped. **D**, PCR screening of knock-in amplicon in C2C12 cells transfected with indicated combinations of spCas9, sgRNA and HDR template. Expected size: WT=N/A; KI=3.1kb. Full length gel images are displayed. **E**, Chromatogram depicting sequence of boundaries between *top:* genomic DNA (gDNA) and left homology arm (HA-L) and *bottom:* HA-L and IRES-GFP. **F**, Fluorescence analysis of Cas9-mCherry and *Gdf11*-IRES-GFP expression in targeted C2C12 cells. Images were taken at 20X magnification.

**
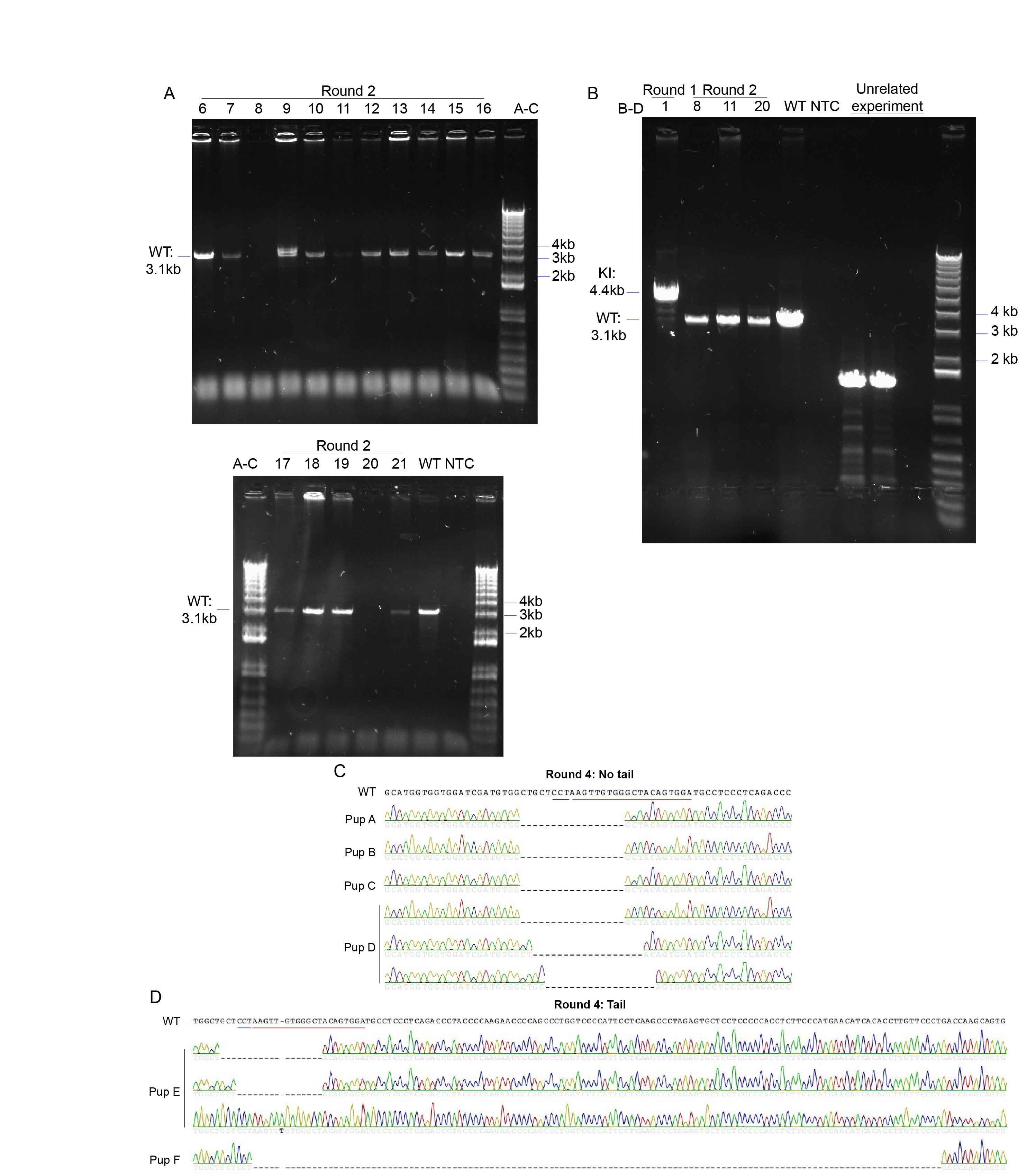
Supplementary Figure 2.** Validation of CRISPR/Cas9-mediated targeting of an IRES-GFP reporter to the *Gdf11* locus in C57BL/6J mice. **A**, PCR screening of founder mice from Round #2 of injections using primer pair A-C. Expected size: WT=3.1kb; KI=4.3kb. NTC: No template control. Full length gel images are displayed. The WT and NTC controls for the PCR were run on the bottom gel. **B**, PCR screening of a subset of founder mice from Rounds #1 and #2 using primer pair B-D, where indicated. Expected size: WT=3.1kb; KI=4.4kb. NTC: No template control. Full length gel images are displayed. Three lanes were from an unrelated experiment (as indicated). **C-D**, Sequences of *Gdf11* target locus in dead pups obtained from Round #4 of injections that **C**, lacked a tail, and **D**, had a tail.

**
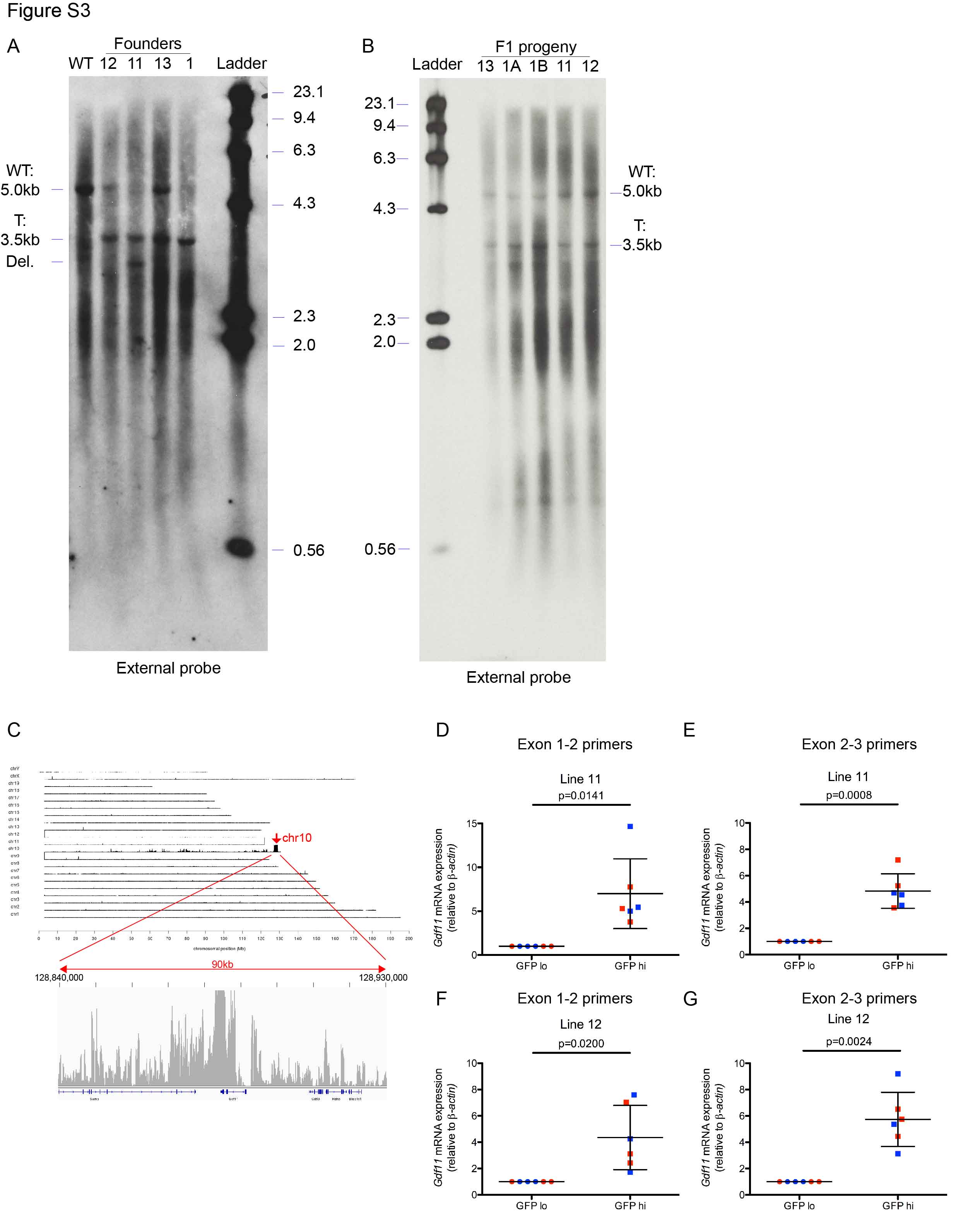
Supplementary Figure 3.** Additional validation of *Gdf11*-IRES-GFP knock-in reporter mouse lines. **A-B**, Southern blot analysis of **A**, *Gdf11*-IRES-GFP targeted founder mice and **B**, *Gdf11*-IRES-GFP F1 progeny. Nco1-digested genomic DNA was hybridized with the external probe. Expected fragment size: WT=5.0kb; T (targeted)=3.5kb. Del.: Deletion. Full length blots are presented. **C**, TLA sequencing coverage and analysis plots from line 1B using outward facing primers in the right homology arm (HA-R). **D-G**, Real time PCR analysis of *Gdf11* levels in FACS-purified GFP^high^ and GFP^low^ splenocytes from **D-E** line 11 and **F-G** line 12 using **D,F** primers spanning exons 1-2 and **E,G** primers spanning exons 2-3. *β-actin* was used as a housekeeping gene. Transcript levels were normalized to levels in GFP^low^ splenocytes. N=3 males (blue), 3 females (red). Individual data points are overlaid with mean ± SD.

** Supplementary Figure 4.** Incorrectly targeted *Gdf11*-IRES-GFP reporter lines exhibit GFP fluorescence within myeloid cells. **A,** Representative flow cytometry analysis of GFP expression within CD3^+^ T cells, CD19^+^ B cells, CD11b^+^/Ly6G^-^ monocytes and CD11b^+^/Ly6G^+^ neutrophils from peripheral blood. **B,** Quantification of GFP+ T cells, B cells, monocytes and neutrophils in 2-month old mice from lines 1A and 13. N=3-5 males and 3-8 females per genotype. Circles: males. Triangles: Females. Individual data points are overlaid with mean ± SD.

**
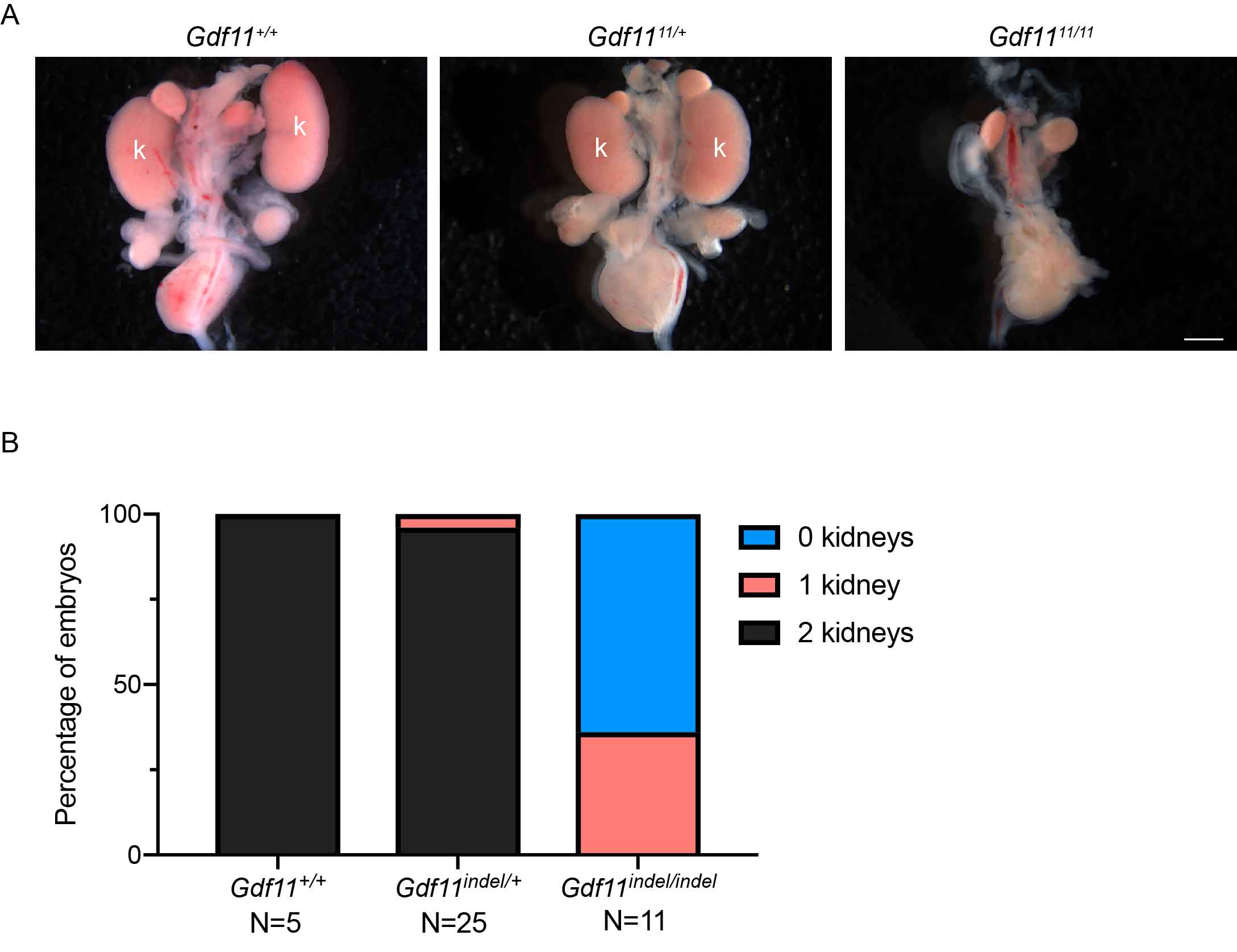
**

**Supplementary Figure 5.** Novel *Gdf11* deletion alleles recapitulate kidney defects observed in *Gdf11* knockout mice. **A**, Representative images of urogenital tracts isolated from E18.5 *Gdf11^+/+^, Gdf11^11/+^, and Gdf11^11/11^* embryos. Note the absence of kidneys in the *Gdf11^11/11^* embryo. Scale bar: 1mm. k: kidney. **B**, Percentage of analyzed embryos exhibiting 0, 1 or 2 kidneys. Quantification was performed in *Gdf11^+/+^*, *GDF11^indel/+^*, and *Gdf11^indel/indel^* E18.5 embryos isolated from lines 4A and 11.

**
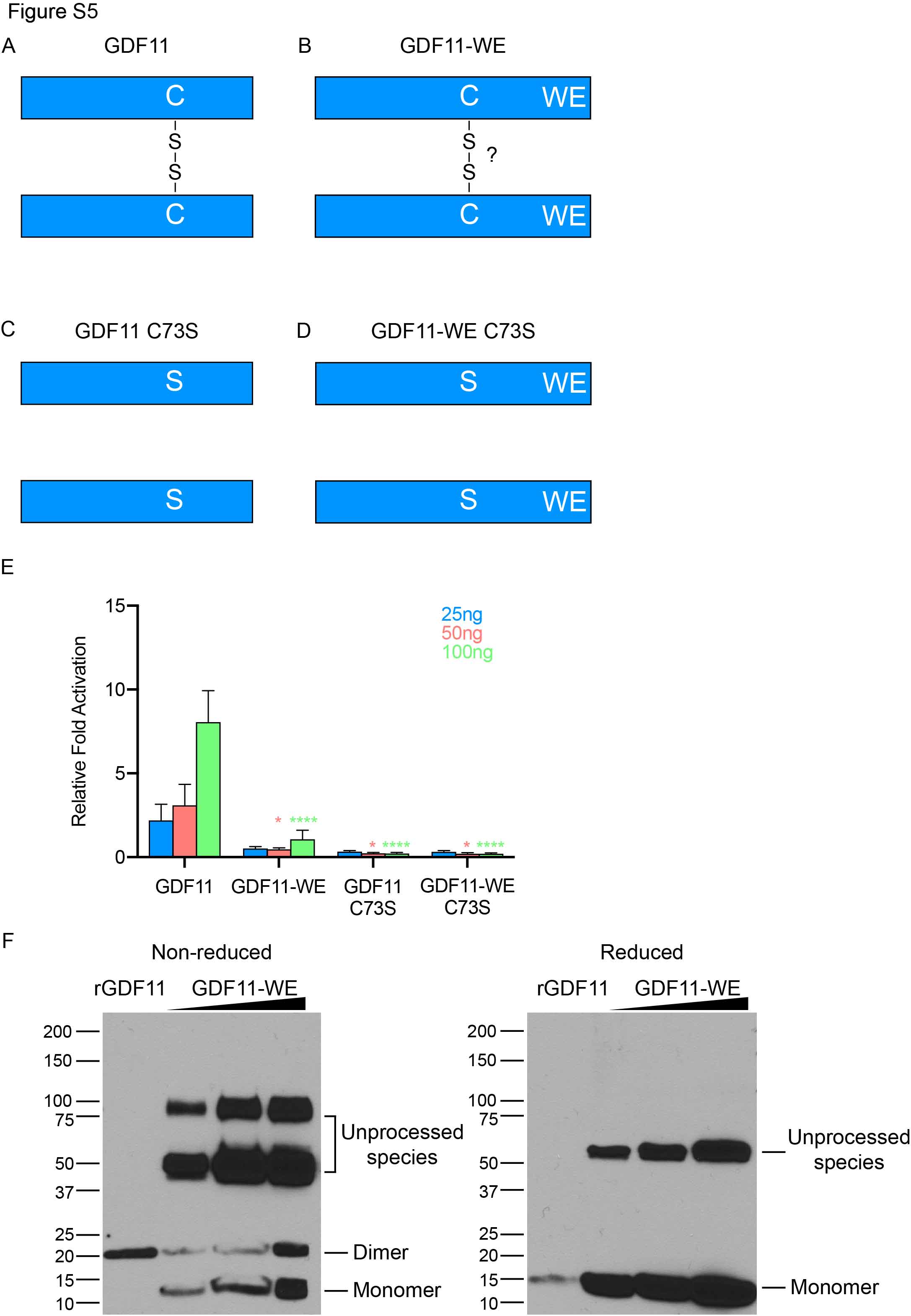
Supplementary Figure 6.** GDF11-WE exhibits impaired signaling capacity in a ((CAGA)_12_ promoter) luciferase reporter gene assay. **A-D,** Schematics of (A) wild-type GDF11, (B) GDF11-WE, (C) the GDF11 C73S mutant, and (D) the GDF11-WE C73S mutant. Wild-type GDF11 monomers form a disulfide bond at the C73 residues, while the C73S mutants do not. The efficiency with which the GDF11-WE variant forms a disulfide bond at the C73 residue is not clear, as denoted by the question mark. **E,** Relative fold activation of luciferase activity in HEK293 cells following transient transfection with 25ng, 50ng, or 100ng of GDF11 WT, GDF11-WE, GDF11 WT C73S, or GDF11-WE C73S. All constructs consist of the human wild-type GDF8 prodomain fused to the wild-type, variant, or mutated mature GDF11 ligand as previously described^58,60^. Use of the GDF8 prodomain serves simply to enhance recombinant ligand production in this system^58,60^. Data are plotted as mean ± SEM from four independent experiments, and each experiment contained 3 technical replicates for each concentration. *p<0.05; ****p<0.0001. P value indications are color-matched to indicate comparison of cells transfected with variant or mutated GDF11 constructs to cells transfected with the equivalent amount of plasmid encoding wild-type GDF11 (leftmost set of columns). **F**, Western analysis of 10ng recombinant GDF11 protein (rGDF11) and increasing amounts of conditioned media from HEK293 cells transfected with GDF11-WE. Both images originate from the same blot. Full length blots are displayed.

**
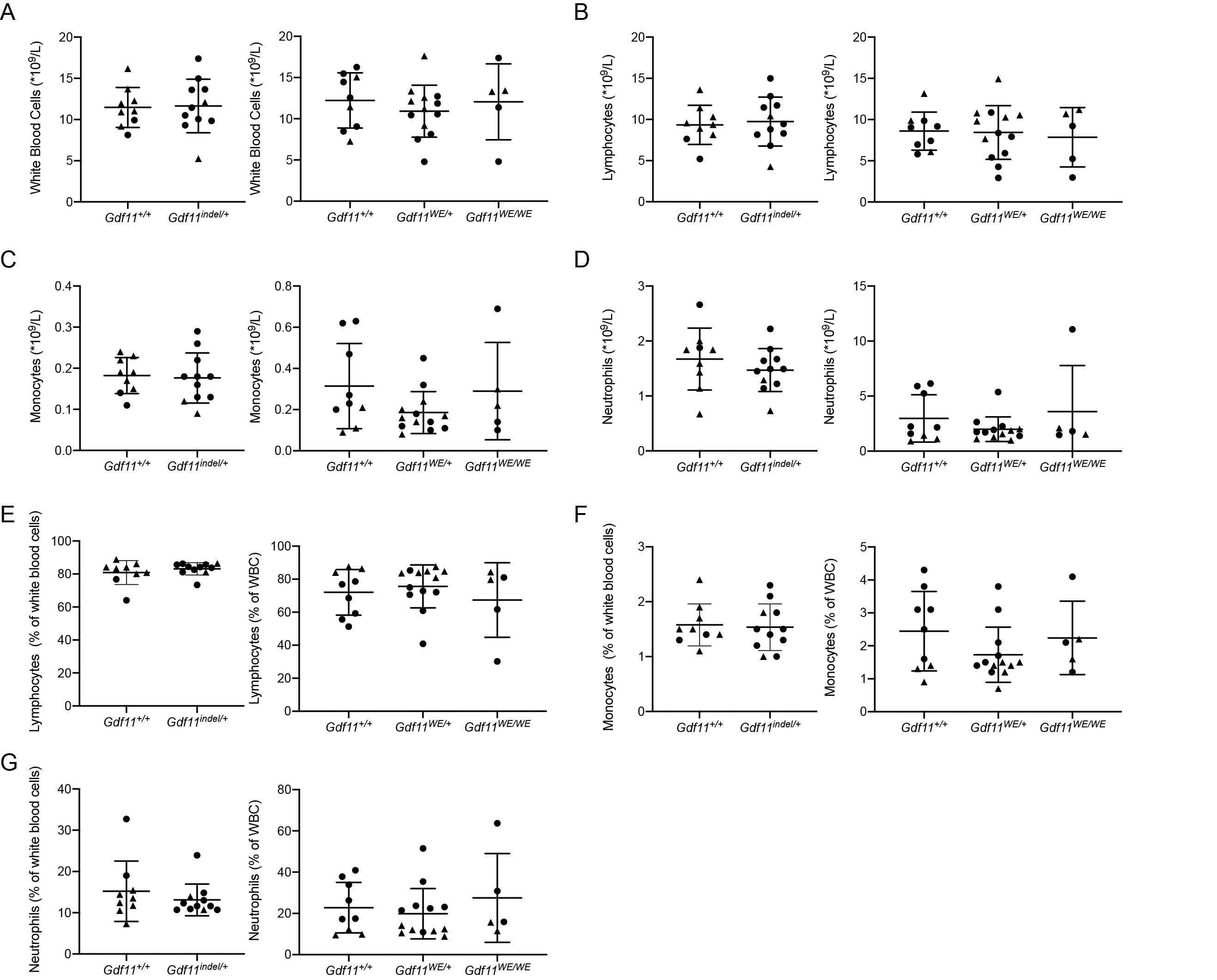
**

**Supplementary Figure 7.** Adult mice containing *Gdf11* deletion alleles do not exhibit altered immune cell abundance or frequency. **A-G**, White blood cell parameters in peripheral blood from 2-4 month old mice. Quantification of (A) white blood cell counts, (B) lymphocyte counts, (C) monocyte counts, (D) neutrophil counts, (E) lymphocyte frequency, (F) monocyte frequency, and (G) neutrophil frequency. For each panel, graphs on the left show wild-type or heterozygous littermates from lines 4A, 4B, and 11 pooled by *Gdf11* genotype (N=2-9 males per genotype; N=2-7 females per genotype); homozygosity for the indels in these lines is not compatible with life. Graphs on the right show *Gdf11* wild-type, heterozygous or homozygous littermates from line 7 containing the GDF11-WE variant (N=3-7 males per genotype; N=2-6 females per genotype). Circles: males. Triangles: females. Individual data points are overlaid with mean ± SD.

**
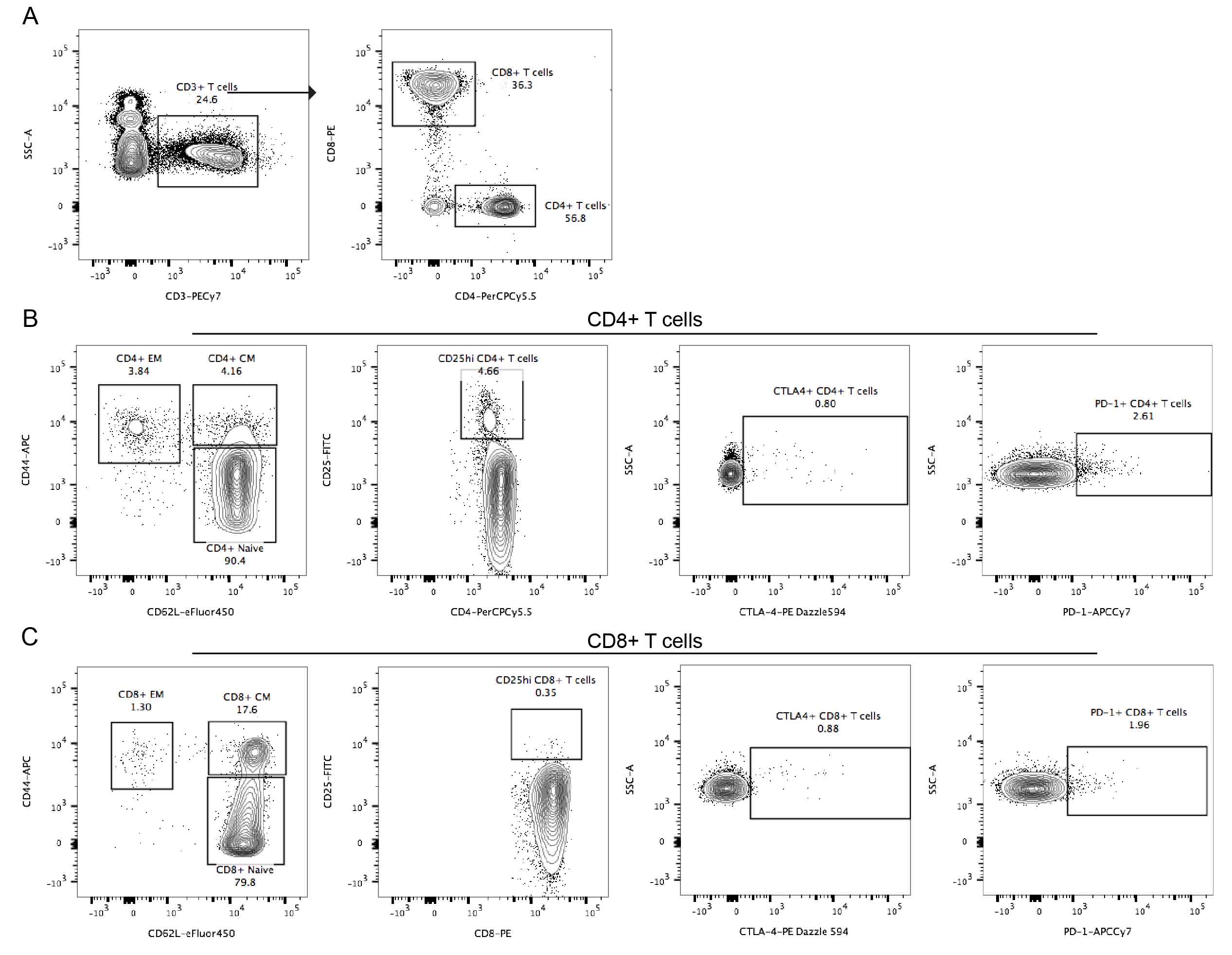
Supplementary Figure 8.** Gating strategy for T cell subsets and T cell activation markers in adult mice with *Gdf11* deletion alleles. **A-C**, Representative flow cytometry analyses of T cell subsets and immune cell activation markers in peripheral blood cells in 3-5 month old mice with *Gdf11* deletion alleles. Analyses include (A) CD3+, CD4+, and CD8+ cells, (B-C) Central Memory (CM), Effector Memory (EM), and Naïve CD4+ and CD8+ T cells, as well as CD4+ and CD8+ T cells expressing CD25 (CD25_hi_), CTLA-4 or PD-1. CD3+ T cells previously gated on live (Zombie Aqua-) singlets.

**
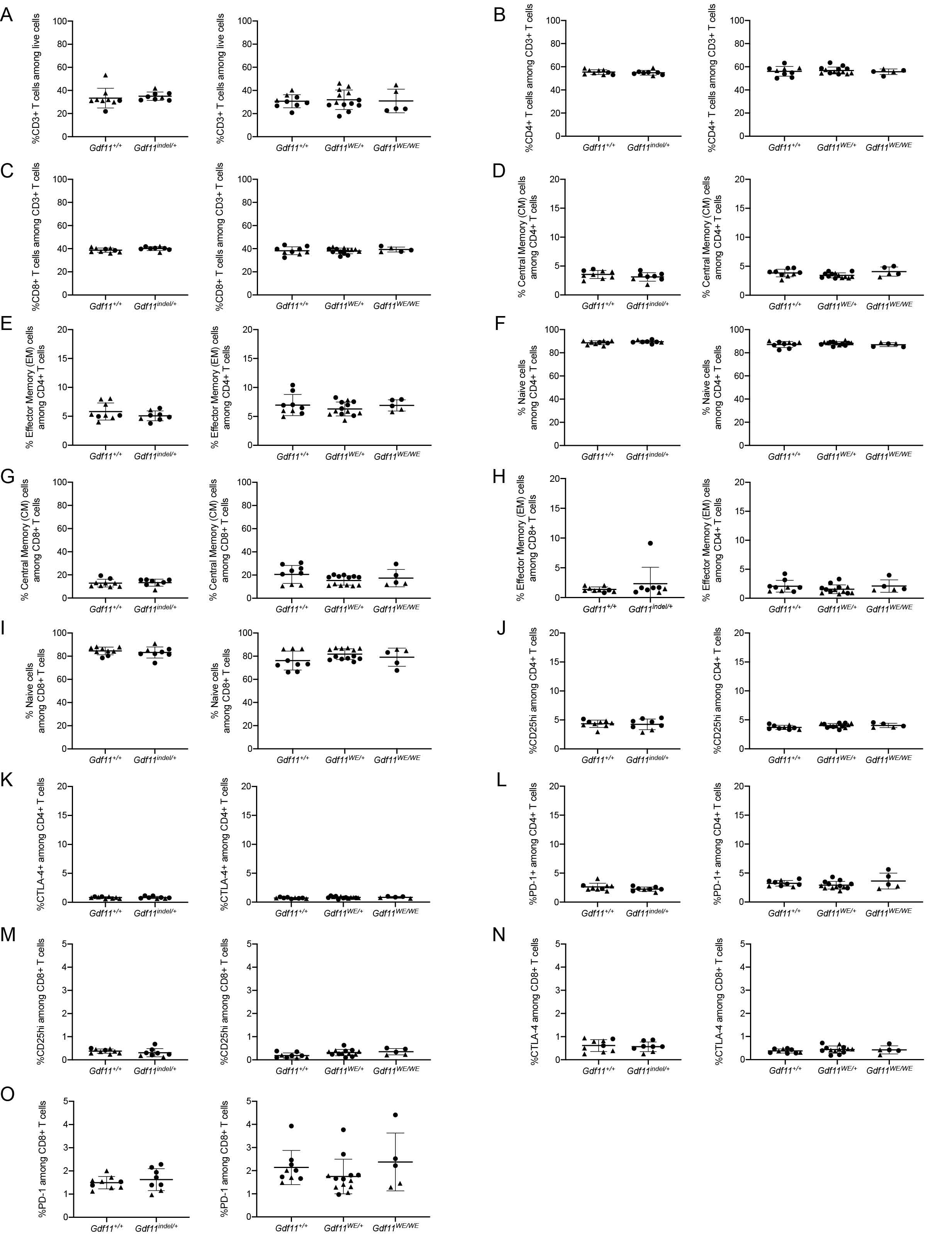
Supplementary Figure 9.** No differences in frequency of T cell subsets or expression of T cell activation markers in adult mice with *Gdf11* deletion alleles. **A-O**, Frequency of (A) CD3+ T cells, (B) CD4+ T cells, (C) CD8+ T cells, (D) CD4+ Central Memory T cells, (E) CD4+ Effector Memory T cells, (F) CD4+ Naïve T cells, (G) CD8+ Central Memory T cells, (H) CD8+ Effector Memory T cells, (I) CD8+ Naïve T cell, (J) CD25hi CD4+ T cells, (K) CTLA-4+ CD4+ T cells, (L) PD-1+ CD4+ T cells, (M) CD25hi CD8+ T cells, (N) CTLA-4+ CD8+ T cells, and (O) PD-1+ CD8+ T cells, within peripheral blood. For each panel, graphs on the left show 3-5 month old mice from lines 4A, 4B, and 11, pooled by genotype (N=2-6 males per genotype; N=2-7 females per genotype); graphs on the right show 3-5 month old mice from line 7 containing the GDF11-WE variant (N=3-7 males per genotype; N=2-6 females per genotype). Circles: males. Triangles: females. Individual data points are overlaid with mean ± SD.

**
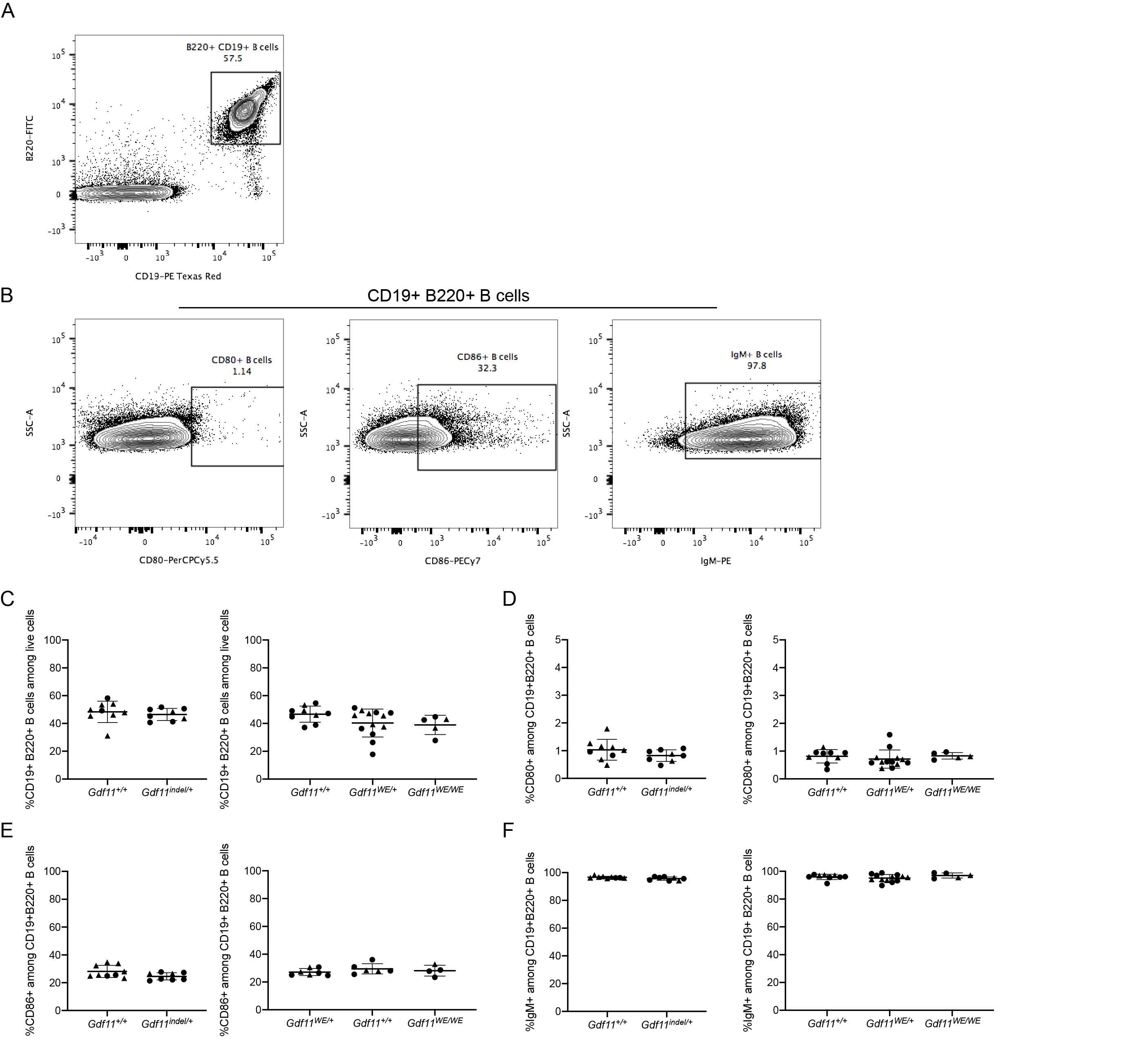
**

**Supplementary Figure 10.** No differences in frequency of B cells or expression of B cell activation markers in adult mice with *Gdf11* deletion alleles. **A-B**, Representative flow cytometry analyses of B cells and B cell activation markers in peripheral blood cells in mice with the indicated *Gdf11* alleles. Analyses include (A) CD19+ B220+ cells, and (B) CD19+ B220+ B cells expressing CD80, CD86 or IgM. CD19+ B220+ B cells previously gated on live (Zombie Aqua-) singlets. **C-F**, Frequency of (C) CD19+ B220+ B cells, (D) CD80+ B cells, (E) CD86+ B cells, and (F) IgM+ B cells within peripheral blood. For each panel, graphs on the left show 3-5 month old mice from lines 4A, 4B, and 11 pooled by genotype (N=2-6 males per genotype; N=2-7 females per genotype); graphs on the right show 3-5 month old mice from line 7 containing the GDF11-WE variant (N=3-7 males per genotype; N=1-6 females per genotype). Circles: males. Triangles: females. Individual data points are overlaid with mean ± SD.

**
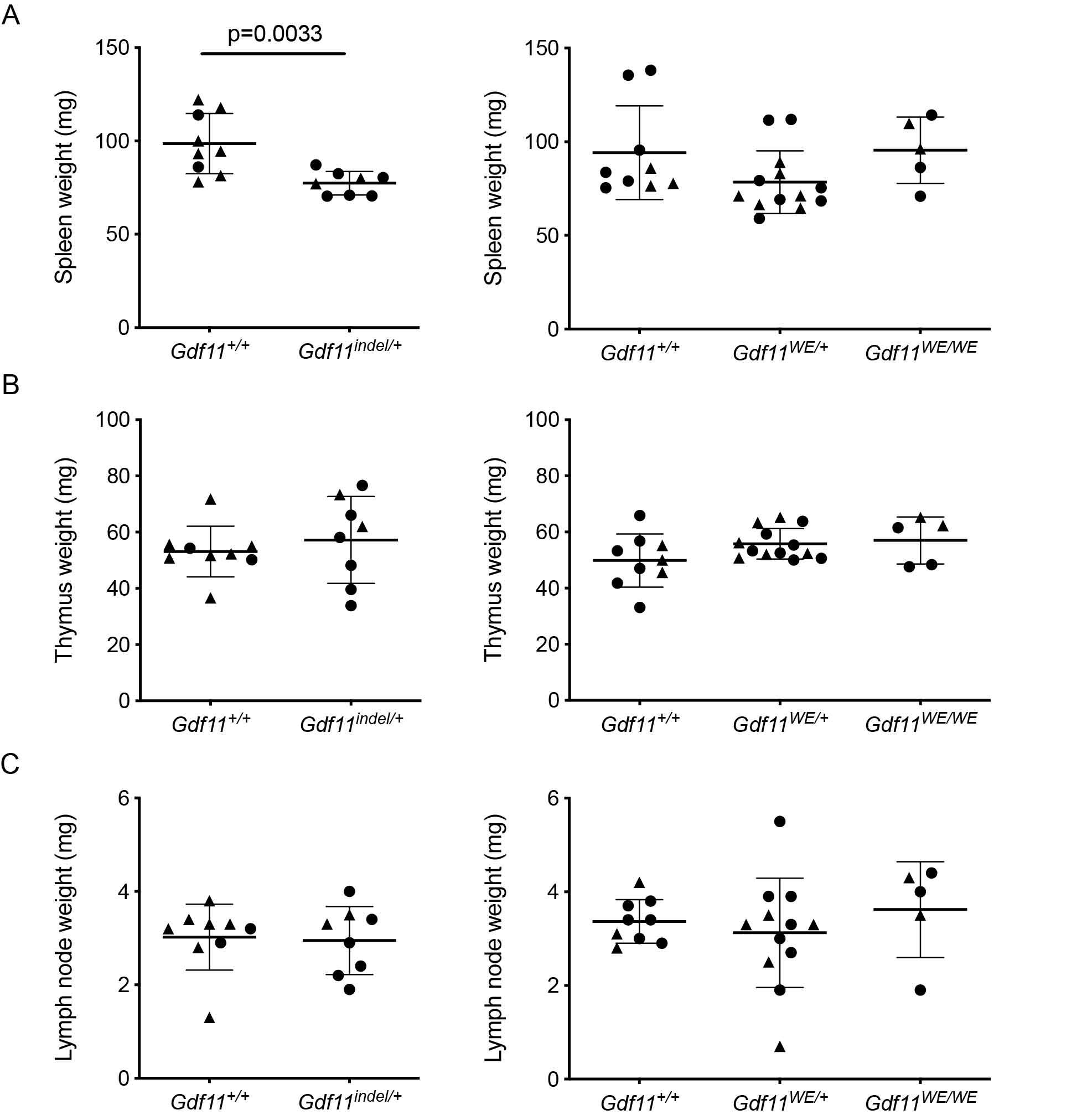
**

**Supplementary Figure 11.** Analysis of immune organ weights in adult mice with *Gdf11* deletion alleles. **A-C**, Analysis of (A) spleen weight, (B) thymus weight, and (C) inguinal lymph node weight in 3-5 month old mice carrying the indicated *Gdf11* genotype. For each panel, graphs on the left show mice from lines 4A, 4B, and 11 pooled by genotype (N=2-6 males per genotype; N=2-7 females per genotype); graphs on the right show mice from line 7 containing the GDF11-WE variant (N=3-7 males per genotype; N=2-6 females per genotype). Circles: males. Triangles: females. Individual data points are overlaid with mean ± SD.

**
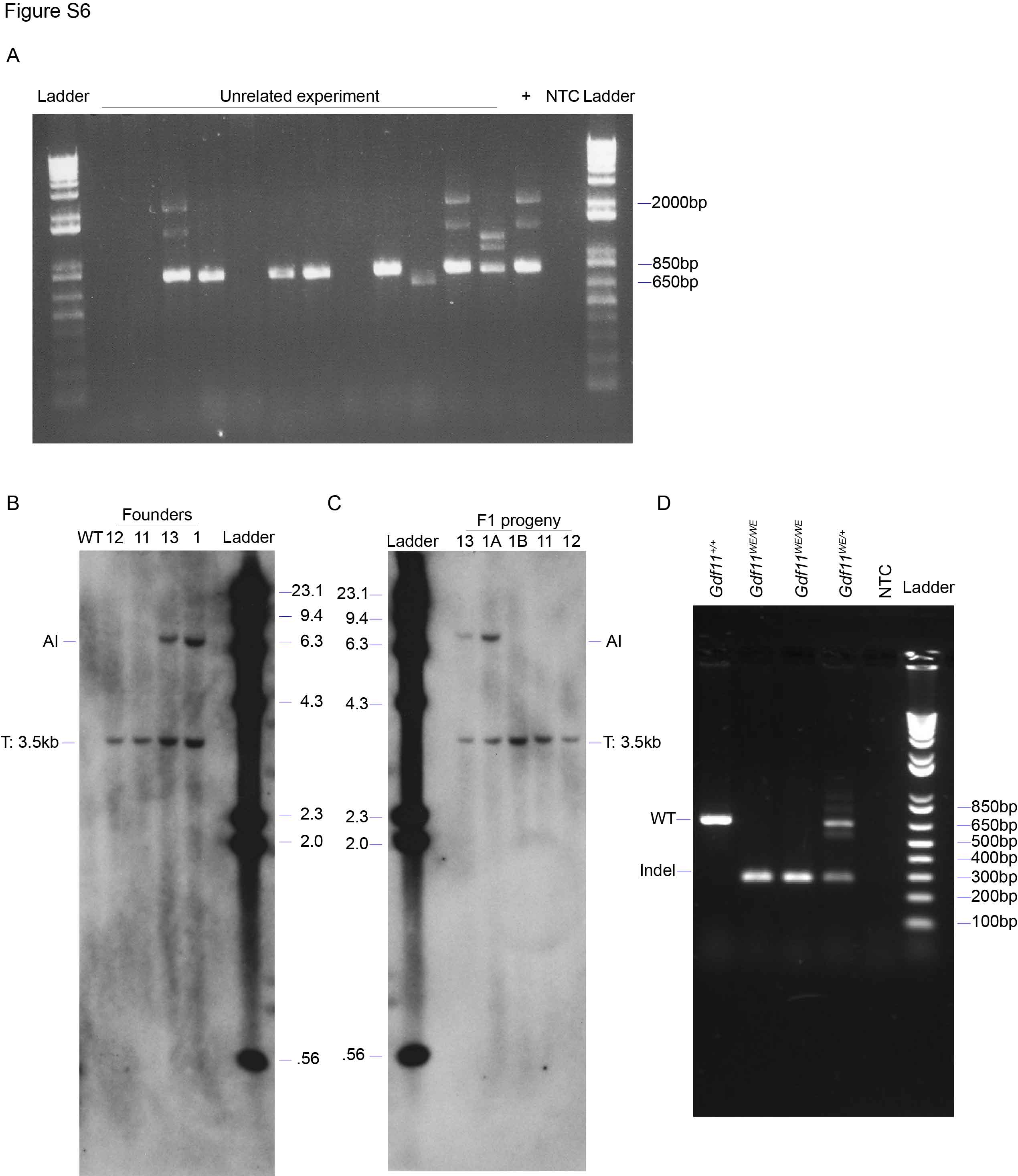
Supplementary Figure 12.** Uncropped Southern blot and agarose gel images. (A) Uncropped agarose gel containing PCR positive (+) control (*Gdf11*-IRES-GFP mouse from line #1), NTC (no template control). Twelve additional lanes on this gel (as indicated) were from an unrelated experiment. (B-C) Uncropped Southern blots from corresponding images in Fig. 2A-B. (D) Uncropped agarose gel image from corresponding image in Fig. 5F.

**Table S1: Analysis of serum cytokines in mice containing *Gdf11* deletion alleles.** Units are in pg/mL. Results are displayed as mean ± standard deviation. Parameters with p<0.05 are bolded.

|  | **Lines 4A, 4B, and 11** | | | **Line 7 (GDF11-WE variant)** | | | |
| --- | --- | --- | --- | --- | --- | --- | --- |
| **Cytokine** | *Gdf11^+/+^*  (N=9) | *Gdf11^indel/+^*  (N=11) | p-value | *Gdf11^+/+^*  (N=9) | *Gdf11^WE/+^*  (N=13)_ | *Gdf11^WE/WE^*  (N=5) | p-value |
| Eotaxin | 612.5 ± 261.5 | 511.3 ± 146.3 | 0.29 | 483.9 ± 85.6 | 559.2 ± 190.1 | 478.3 ± 83.9 | 0.41 |
| G-CSF | 328.9 ± 256.7 | 181.8 ± 70.3 | 0.08 | 249.4 ± 21.3 | 287.6 ± 94.0 | 253.9 ± 126.0 | 0.69 |
| GM-CSF | 0.02 ± 0 | 0.02 ± 0 | n/a | 0.02 ± 0 | 5.836 ± 20.97 | 0.02 ± 0 | 0.60 |
| IFN-γ | 3.66 ± 8.81 | 0.56 ± 0.54 | 0.26 | 1.30 ± 2.97 | 8.97 ± 30.87 | 0.68 ± 0.89 | 0.65 |
| IL-1α | 859.5 ± 484.7 | 755.1 ± 203.8 | 0.52 | 629.1 ± 192.9 | 717.1 ± 207.8 | 686.6 ± 215.3 | 0.62 |
| IL-1β | 1.42 ± 1.37 | 1.18 ± 1.29 | 0.69 | 1.76 ± 2.40 | 2.60 ± 2.65 | 5.79 ± 6.32 | 0.13 |
| **IL-2** | **34.08 ± 35.44** | **6.02 ± 4.29** | **0.02** | 26.54 ± 34.57 | 33.86 ± 77.63 | 4.50 ± 4.77 | 0.64 |
| IL-3 | 0.18 ± 0.14 | 0.30 ± 0.31 | 0.28 | 0.21 ± 0.29 | 0.43 ± 0.62 | 0.16 ± 0.25 | 0.43 |
| IL-4 | 0.07 ± 0.07 | 0.18 ± 0.23 | 0.16 | 0.10 ± 0.08 | 0.30 ± 0.82 | 0.84 ± 1.37 | 0.27 |
| IL-5 | 7.92 ± 4.45 | 6.23 ± 11.20 | 0.68 | 4.68 ± 3.72 | 5.15 ± 3.15 | 6.99 ± 6.60 | 0.59 |
| IL-6 | 5.85 ± 12.49 | 0.97 ± 2.33 | 0.22 | 17.95 ± 19.96 | 8.22 ± 9.44 | 16.77 ± 32.52 | 0.45 |
| IL-7 | 0.27 ± 0.74 | 18.73 ± 39.35 | 0.18 | 0.55 ± 1.48 | 47.11 ± 124.2 | 2.66 ± 5.91 | 0.41 |
| IL-9 | 56.49 ± 116.2 | 25.05 ± 15.75 | 0.38 | 39.95 ± 34.78 | 78.23 ± 211.2 | 26.26 ± 6.45 | 0.75 |
| IL-10 | 0.82 ± 0.64 | 0.91 ± 0.91 | 0.79 | 1.02 ± 0.66 | 8.90 ± 29.80 | 1.17 ± 0.71 | 0.63 |
| IL-12 (p40) | 1.05 ± 1.26 | 1.50 ± 4.19 | 0.76 | 0.20 ± 0.54 | 7.60 ± 25.42 | 0.02 ± 0 | 0.57 |
| IL-12 (p70) | 0.02 ± 0 | 0.77 ± 2.49 | 0.38 | 0.31 ± 0.87 | 28.87 ± 104.0 | 4.31 ± 9.60 | 0.64 |
| IL-13 | 20.65 ± 15.00 | 10.93 ± 5.03 | 0.06 | 12.89 ± 5.67 | 32.85 ± 66.27 | 10.68 ± 2.82 | 0.53 |
| IL-15 | 0.21 ± 0.37 | 37.66 ± 124.6 | 0.38 | 0.52 ± 1.51 | 109.2 ± 386.2 | 0.02 ± 0 | 0.59 |
| IL-17A | 0.21 ± 0.27 | 0.22 ± 0.31 | 0.90 | 0.34 ± 0.45 | 0.73 ± 1.20 | 0.93 ± 1.22 | 0.52 |
| IP-10 | 80.48 ± 42.44 | 61.71 ± 53.36 | 0.40 | 63.33 ± 18.21 | 67.87 ± 33.89 | 62.72 ± 17.31 | 0.90 |
| KC | 154.6 ± 165.0 | 92.33 ± 69.02 | 0.27 | 135.3 ± 99.5 | 210.6 ± 279.6 | 109.5 ± 77.8 | 0.57 |
| LIF | 0.93 ± 2.73 | 0.55 ± 1.65 | 0.70 | 0.70 ± 1.32 | 0.88 ± 3.05 | 0.02 ± 0 | 0.78 |
| MCP-1 | 14.41 ± 6.69 | 14.20 ± 11.34 | 0.96 | 18.72 ± 20.48 | 18.19 ± 6.62 | 34.58 ± 52.49 | 0.44 |
| **M-CSF** | **1.22 ± 0.89** | **2.44 ± 1.43** | **0.04** | 2.85 ± 3.71 | 3.55 ± 7.23 | 22.24 ± 1.38 | 0.89 |
| MIG | 14.46 ± 12.86 | 7.19 ± 3.23 | 0.09 | 5.99 ± 2.19 | 8.58 ± 3.66 | 8.88 ± 3.74 | 0.15 |
| MIP-1α | 40.84 ± 70.55 | 12.84 ± 22.18 | 0.23 | 100.4 ± 203.7 | 81.40 ± 226.6 | 21.58 ± 30.52 | 0.77 |
| MIP-1β | 38.56 ± 66.20 | 9.45 ± 31.29 | 0.21 | 31.37 ± 89.27 | 36.77 ± 79.30 | 0.02 ± 0 | 0.65 |
| MIP-2 | 5.18 ± 8.45 | 5.00 ± 7.54 | 0.82 | 2.97 ± 4.65 | 8.55 ± 14.34 | 1.50 ± 2.78 | 0.33 |
| RANTES | 41.28 ± 32.91 | 40.62 ± 29.98 | 0.96 | 20.06 ± 16.30 | 35.58 ± 23.01 | 22.22 ± 14.37 | 0.17 |
| TNFα | 0.33 ± 0.93 | 0.02 ± 0 | 0.28 | 0.10 ± 0.25 | 0.16 ± 0.52 | 0.02 ± 0 | 0.78 |
| VEGF | 0.88 ± 1.28 | 0.38 ± 0.11 | 0.21 | 1.00 ± 0.81 | 2.74 ± 7.87 | 0.63 ± 0.51 | 0.68 |

**Table S2: Semi-quantitative analysis of Ig sub-classes and Ig light chain types in serum from mice containing *Gdf11* deletion alleles.** Values are normalized fluorescence levels (arbitrary units). Results are displayed as mean ± standard deviation. No parameters exhibited p<0.05.

|  | **Lines 4A, 4B, and 11** | | | **Line 7 (GDF11-WE variant)** | | | |
| --- | --- | --- | --- | --- | --- | --- | --- |
| **Ig** | *Gdf11^+/+^*  (N=9) | *Gdf11^indel/+^*  (N=11) | p-value | *Gdf11^+/+^*  (N=9) | *Gdf11^WE/+^*  (N=11) | *Gdf11^WE/WE^*  (N=5) | p-value |
| IgA | 6,229 ± 1,539 | 7,146 ± 1,527 | 0.20 | 8,232 ± 3,483 | 9,388 ± 4,904 | 9,190 ± 2,695 | 0.81 |
| IgD | 987.6 ± 120.5 | 1,043 ± 356.4 | 0.66 | 1,126 ± 393.6 | 986.6 ± 339.6 | 972.8 ± 176.8 | 0.60 |
| IgE | 708.8 ± 167.9 | 636.7 ± 129.0 | 0.29 | 798.9 ± 137.4 | 811.2 ± 167.8 | 676.1 ± 80.68 | 0.22 |
| IgM | 41,935 ± 19,256 | 32,793 ± 14,541 | 0.24 | 47,339 ± 12,350 | 37,349 ± 12,022 | 49,326 ± 16,764 | 0.15 |
| IgG1 | 50,060 ± 33,905 | 39,043 ± 27,154 | 0.43 | 70,051 ± 33,376 | 48,822 ± 28,531 | 69,191 ± 15,787 | 0.22 |
| IgG2a | 24,780 ± 20,260 | 27,713 ± 22,452 | 0.77 | 58,514 ± 41,854 | 48,977 ± 34,402 | 79,379 ± 37,261 | 0.35 |
| IgG2b | 84,862 ± 82,760 | 56,972 ± 72,728 | 0.44 | 139,848 ± 80,859 | 120,748 ± 108,990 | 159,692 ± 111,308 | 0.76 |
| IgG3 | 2,761 ± 1,028 | 3,240 ± 2,200 | 0.56 | 5,044 ± 1,589 | 3,649 ± 2,178 | 5,373 ± 1,565 | 0.15 |
| Lambda | 21,608 ± 9,809 | 23,215 ± 16,713 | 0.80 | 32,112± 8,386 | 28,066 ± 11,570 | 31,828 ± 7,141 | 0.61 |
| Kappa | 200,228 ± 71,117 | 188,131 ± 104,591 | 0.77 | 256,878 ± 54,340 | 235,328 ± 71,070 | 234,199 ± 51,869 | 0.70 |

**Table S3: sgRNA target sequences**

*Gdf11* spCas9 sgRNA targets

| **Name** | **Sequence (5’-3’)** |
| --- | --- |
| *Gdf11*_sgRNA1 | ATGTGGCTGCTCCTAAGTTG |
| *Gdf11*_sgRNA2 | TGTGGCTGCTCCTAAGTTGT |
| *Gdf11*_sgRNA3 | TCCACTGTAGCCCACAACTT |
| *Gdf11*_sgRNA4 | TCCTAAGTTGTGGGCTACAG |

**Table S4: Oligonucleotides used in this study**

Oligonucleotides used for production of sgRNA template for *in vitro* transcription

| **Template** | **Direction** | **Sequence (5’-3’)** |
| --- | --- | --- |
| *Gdf11*_sgRNA3 | F | TTAATACGACTCACTATAGGGTCCACTGTAGCCCACAACTT |
|  | R | AAAAGCACCGACTCGGTGCC |

Oligonucleotides used for Gibson Assembly of HDR template

| **Template** | **Direction** | **Sequence (5’-3’)** |
| --- | --- | --- |
| Left Homology Arm_nested PCR_external amplicon | F | ATGCAGATGGTAATACTTGGG |
|  | R | GTTCATGGGAAGAGGTGGGG |
| Left Homology Arm_nested PCR_internal amplicon | F | GCAAACAAAGGGAGGTCAAGG |
|  | R | AAGCGGCTTCGGCCAGTAACGTTAGCTTAAGAGCAGCCACATCGA |
| IRES-GFP | F | CTAACGTTACTGGCCGAAGC |
|  | R | TTACTTGTACAGCTCGTCCA |
| Right Homology Arm_nested PCR_external amplicon | F | CATGCAAAAGTATCCACACACC |
|  | R | CAGGTTCAAGTGCTGGATTG |
| Right Homology Arm_nested PCR_internal amplicon | F | GCATGGACGAGCTGTACAAGTAATTGTGGGCTACAGTGGATGCC |
|  | R | GTTCCTCTCCTACACCAAGA |
| pUC19 backbone | F | TCTTGGTGTAGGAGAGGAACGGCGTAATCATGGTCATAGC |
|  | R | CTTGACCTCCCTTTGTTTGCGTTTTACAACGTCGTGACTGG |

Oligonucleotides used for (a) T7E1 mismatch detection assay and for (b) internal amplicon screening of CRISPR-mediated HDR of *Gdf11*-IRES-GFP template in F_0_ mice

| **Gene Target** | **Direction** | **Sequence (5’-3’)** |
| --- | --- | --- |
| *Gdf11* (primer B) | F | CCTGGACTGCGATGAACACT |
| *Gdf11* (primer C) | R | CCTACTGAGTTGTCAGGGGAAC |

Oligonucleotides used to evaluate CRISPR-mediated HDR of *Gdf11*-IRES-GFP template in C2C12 cells

| **Gene Target** | **Direction** | **Sequence (5’-3’)** |
| --- | --- | --- |
| *Gdf11*_ semi-nested PCR_external amplicon | F | ATGCAGATGGTAATACTTGGG |
|  | R | CCTACTGAGTTGTCAGGGGAAC |
| *Gdf11*_ semi-nested PCR_internal amplicon | F | ATGCAGATGGTAATACTTGGG |
|  | R | TTCAAAGGAAAACCACGTCC |

Oligonucleotides used to evaluate CRISPR-mediated HDR of *Gdf11*-IRES-GFP template in F_0_ mice

| **Gene Target** | **Direction** | **Sequence (5’-3’)** |
| --- | --- | --- |
| *Gdf11* (primer A) | F | ATGCAGATGGTAATACTTGGG |
| *Gdf11* (primer C) | R | CCTACTGAGTTGTCAGGGGAAC |
| *Gdf11* (primer B) | F | CCTGGACTGCGATGAACACT |
| *Gdf11* (primer D) | R | CAGGTTCAAGTGCTGGATTG |

Oligonucleotides used to synthesize DIG-labelled probes for Southern blot analysis

| **Gene Target** | **Direction** | **Sequence (5’-3’)** |
| --- | --- | --- |
| *GFP*_internal probe | F | GGACGACGGCAACTACAAGA |
|  | R | TTACTTGTACAGCTCGTCCA |
| *Gdf11*_external probe | F | TGGGGGAAGGGGTTGTCTTA |
|  | R | CAGGTTCAAGTGCTGGATTG |

Outward facing oligonucleotides used for TLA sequencing

| **Template** | **Direction** | **Sequence (5’-3’)** |
| --- | --- | --- |
| Primer set 1 (within GFP) | F | GAGAAGCGCGATCACATG |
|  | R | GTGTTCTGCTGGTAGTGG |
| Primer set 2 (within right homology arm) | F | ACCAATCTACTTAAGCACTTGT |
|  | R | CTCCCTCTGTTGTATTGCAC |

Oligonucleotides used for real-time PCR analysis

| **Gene Target** | **Direction** | **Sequence (5’-3’)** |
| --- | --- | --- |
| *Gdf11*_exon 1-2 amplicon | F | CTACCACCGAGACGGTCATAA |
|  | R | CCGAAGGTACACCCACAGTT |
| *Gdf11*_exon 2-3 amplicon | F | ACAGAGCAACTGGGGAATCG |
|  | R | AGTGTTCATCGCAGTCCAGG |
| *β-actin* | F | GGCTGTATTCCCCTCCATCG |
|  | R | CCAGTTGGTAACAATGCCATGT |

Oligonucleotides used for production of *Gdf11* and *GFP* templates to use for *in vitro* transcription of probes for *in situ* hybridization

| **Gene Target** | **Direction** | **Sequence (5’-3’)** |
| --- | --- | --- |
| *Gdf11* | F | CATGCAAAAGTATCCACACACC |
|  | R | taatacgactcactatagggCCTACTGAGTTGTCAGGGGAAC |
| *GFP* | F | CTGGTCGAGCTGGACGGCGACG |
|  | R | taatacgactcactatagggTTACTTGTACAGCTCGTCCA |

Oligonucleotides used for site-directed mutagenesis

| **Gene Target** | **Direction** | **Sequence (5’-3’)** |
| --- | --- | --- |
| GDF11/ WE | F | TGGGTGCTCATGGGAGTAAGACCTGCAGAAGCTTGGC |
|  | R | CTTCTGCAGGTCTTACTCCCATGAGCACCCACAGCGG |
| GDF11/ C73S | F | TCAGCAGGCCCTtcaTGTACTCCCACAAAGATG |
|  | R | TGTGGGAGTACAtgaAGGGCCTGCTGAACCTCT |
